# Statistical normalization methods in microbiome data with application to microbiome cancer research

**DOI:** 10.1080/19490976.2023.2244139

**Published:** 2023-08-25

**Authors:** Yinglin Xia

**Affiliations:** Division of Gastroenterology and Hepatology, Department of Medicine, University of Illinois Chicago, Chicago, USA

**Keywords:** Microbiome, normalization, 16S rRNA sequencing data, shotgun metagenomic sequencing data, microbiome cancer research

## Abstract

Mounting evidence has shown that gut microbiome is associated with various cancers, including gastrointestinal (GI) tract and non-GI tract cancers. But microbiome data have unique characteristics and pose major challenges when using standard statistical methods causing results to be invalid or misleading. Thus, to analyze microbiome data, it not only needs appropriate statistical methods, but also requires microbiome data to be normalized prior to statistical analysis. Here, we first describe the unique characteristics of microbiome data and the challenges in analyzing them (Section 2). Then, we provide an overall review on the available normalization methods of 16S rRNA and shotgun metagenomic data along with examples of their applications in microbiome cancer research (Section 3). In Section 4, we comprehensively investigate how the normalization methods of 16S rRNA and shotgun metagenomic data are evaluated. Finally, we summarize and conclude with remarks on statistical normalization methods (Section 5). Altogether, this review aims to provide a broad and comprehensive view and remarks on the promises and challenges of the statistical normalization methods in microbiome data with microbiome cancer research examples.

## Introduction

The advancement of high-throughput sequencing and next generation sequencing (NGS) techniques, including 16S ribosomal RNA (rRNA) and whole shotgun metagenomic sequencing (WSMS), has promoted microbiome study in cancer research.^1^ It has been shown that the microbiome is associated with various cancers, which not only include gastrointestinal (GI) tract cancers,^2,3^ such as esophageal cancer,^4^ gastric cancer,^5^ colorectal cancers (CRC),^6–8^ pancreatic cancer,^9–11^ liver cancer,^12–14^ but also include non-GI tract cancers such as lung cancer,^15^ breast cancer,^16^ prostate cancer,^17^ melanoma,^18,19^ and epithelial tumors.^20^

However, microbiome data analysis is very challenging because microbiome data have some unique characteristics, which often make the results of standard statistical tests/methods to be invalid or misleading. Thus, microbiome data not only need appropriate statistical methods for analysis, but also require normalization prior to analysis. In this review article, we describe some unique characteristics of microbiome data and the challenges in analysis, provide an overall review on the available normalization methods of 16S rRNA and shotgun metagenomic data with examples in microbiome cancer research. We also comprehensively investigate how the normalization methods of 16S rRNA and shotgun metagenomic data along with their accompanying statistical methods are evaluated.

## Unique characteristics and challenges for statistical analysis of 16S rRNA and shotgun metagenomic data

Microbiome raw sequence data generally are generated via amplicon sequencing and shotgun metagenomic sequencing techniques. The amplicon sequencing approach only amplifies and sequences one particular gene, often 16S ribosome RNA (rRNA) gene, while shotgun metagenomic sequencing approach assays the collective genome from all the microbial species in a given sample. The 16S rRNA sequencing approach has been described with more details else where.^21,22^ Here, we introduce a little bit more on the history of shotgun sequencing method before presenting the unique characteristics of microbiome data.

The shotgun sequencing technique was developed to complement traditional phylotyping approaches such as 16S rRNA sequencing approach and genome sequencing of culturable ecosystem members by combining an environment (the “metagenome” of a community).^23,24^ In 2004, large-scale environmental shotgun sequencing projects were first published.^25–27^ Shotgun metagenomics studies microorganisms or microbial communities by sequencing DNA fragments directly from samples without any prior cultivation of individual isolates.^28^ With advancements in shotgun sequencing technologies, this approach very largely facilitates our efforts to investigate the genetic basis of environmental diversity, promising not only to uncover the identity but also the functionality of the most “unculturable” microbial species on earth, and provide insights into our understanding of ecosystem functioning.^24^ Through using high-throughput sequencing, almost an entire microbial community can be profiled, which enables us to study the unculturable microorganisms in their natural state.^29,30^

Metagenomic analyses have three basic tasks via taxonomic analysis, functional analysis, and comparative analysis to answer three basic questions: 1) who are they 2) what can they do, and 3) how to compare them? Gene-centric metagenomic analysis quantifies the biological functions (“genes”) present in a metagenome. The generated metagenomic data can be organized in a gene expression matrix or a table of gene counts (observed DNA fragments) with N rows and P columns. The N rows are combinedly referred to as genes, corresponding to bins, typically representing genes, including gene families, functional groups, or single genes. The P columns represent metagenomes from different conditions of samples. Typically, the gene count table is generated through several steps.^31,32^ Among them, three steps are critical.^33:^ The first step is to assess the quality of the generated sequence DNA fragments (reads). The second step is to align reads to an annotated reference database. The third step is to estimate or bin the abundance of each gene to match each generated sequence read against an annotated reference database.

Microbiome research has expanded rapidly across diverse fields over the past two decades. However, this extremely fast-growing discipline faces a variety of challenges.^34–37^ In our books and reviewes, we have described some unique characteristics of microbiome data and the challenges in statistical analysis of microbiome data.^22,33–35,38,39^ Microbiome data, including 16S rRNA and metagenomic shotgun sequencing, have six unique characteristics. They are:

1. classified into hierarchical taxonomic ranks and encoded as a phylogenetic tree;
2. multivariate or high dimensional;
3. compositional;
4. over-dispersed;
5. sparse and often have excess zeros (zero-inflated); and
6. heterogeneous.

Shotgun metagenomics and 16S rRNA studies may have slightly different data characteristics.^35,36^ Compared to 16S rRNA study, 1) it has even smaller number of samples; 2) it is more plagued by high levels of biological and technical variability; 3) its zero-inflation is more due to under-sampling; 4) its data characteristics is closer to RNA-seq data; and 5) it has more over-dispersion compared to zero-inflation. However, both shotgun metagenomics and 16S rRNA studies face similar challenges.

The unique structure and characteristics of microbiome data pose major challenges on data integration and statistical analysis. At least four challenges in the statistical analysis of microbiome data can be summarized: 1) high-dimensionality causes the large P and small N problem; 2) tree structure and compositionality cause the dependency problem; 3) sparsity with excess zeros causes the over-dispersion and zero-inflation problems; and 4) heterogeneity challenges data integration, modeling, and meta-analysis.

In summary, 1) the large P and small N, and sparsity problems not only require additional assumptions for accurate inference, but also severely reduce the power for inferencing taxon-taxon or gene-gene association analyses; 2) the compositionality may introduce false positive taxon-taxon or taxa-covariate associations, precluding traditional correlation analysis for the detection of taxon-taxon or gene-gene relationships; and 3) the heterogeneity not only challenges integrating the data, and conducting comparisons of studies and meta-analysis, but also makes interpretability difficult.

## Normalization methods for 16S rRNA and shotgun metagenomic data

It is now commonly accepted that preprocessing a normalization step can significantly improve the quality of the downstream analysis, in particular, for the differential gene expression analysis of RNA-seq data and the differential abundance analysis of microbiome data. Various systemic biases exist in microbiome data that impact detecting biological variations between samples, such as variations in sample collection, library preparation, and sequencing process, i.e., uneven sampling depth and sparsity. Normalization is expected to mitigate some of these artifactual biases in the original measurements to enable downstream analysis to accurately compare biological differences. The choice of different normalization methods often leads to a very different result in terms of differential gene expression or microbial abundance analysis. Nevertheless, which normalization method is more appropriate to the fitting data is still arguable.

In early microbiome literature, most normalization and associated statistical methods were adopted from other relevant research fields such as RNA-sequencing and ecology. Around twelve years ago, the term “differential abundance” was coined as a direct analogy to differential expression from RNA-Seq and has been adopted for use in microbiome literature.^40–42^ Currently, several normalization methods have been developed for 16S rRNA microbiome data, while specifically designed normalization methods for shotgun metagenomics data are rare. Most normalization methods used in shotgun metagenomics data are adopted from other fields of high-dimensional count data, such as RNA-seq data. As a foundation for quantitative analysis, Beszteri et al.^43^ and Frank and Sørensen^44^ have evaluated the normalization method, “average genome size”, for metagenomics data. Similar to RNA-seq data, after initial quality control steps to account for errors in the sequencing process, microbial community sequencing data is typically organized into a so-called “OTU-table (matrix)” or “ASV-table (matrix)” or feature-table, in which each row presents the OTU (observed counts of clustered sequences: taxon or bacteria types), each column presents the sample (or library), and each cell presents the number of OTU (or taxon) read counts mapped to the OTU (taxon) in the sample. Data normalization and differential abundant analysis typically start with the “OTU-table (matrix)”. Similar to RNA-seq data and 16S rRNA-seq data, normalization in shotgun metagenomic data is the processing of the count matrix of gene abundance data, with each row presenting samples and each column presenting genes. The read counts describe the number of DNA fragments sampled for each gene from a microbial community.

Most normalization methods that are currently used in 16S rRNA-seq and shotgun metagenomic data are adopted from RNA-seq data, which can be summarized into four categories, based on technical or statistical approach as well as the kinds of data that these methods originally target to normalize (Table 1): 1) Ecology data-based normalization methods. 2) Traditional normalization methods. 3) RNA-seq data-based normalization methods. 4) Microbiome data-based normalization methods, including a) mitigating over-dispersion normalization; b) mitigating zero-inflation normalization; c) compositionally aware normalization; and d) hybrid-based normalization methods. We remark on the normalization methods for shotgun metagenomic data separately when they are different from the use in 16S rRNA data.

**Table 1.** Normalization methods in 16S rRNA and shotgun metagenomic data.

Method	Definition/Procedure
**Ecology data-based normalization methods**	
Rarefying.^40,45,46^ Based on hypergeometric model.^47^	Subsampling each column of sequences to an even depth without replacement, i.e., randomly removes reads in the different samples until all samples reach the same predefined number (usually set to the lowest sample size among all samples in the dataset) of reads, thereby assuring a uniform sequence depth. Rarefying most often consists of the following three steps: 1) select a minimum library size, 2) discard libraries (microbiome samples) that have fewer reads than the predefined minimum library size, and 3) subsample the remaining libraries, without replacement, until all libraries have the minimum size.^48,49^
**Traditional normalization methods**	
Total Sum Scaling (TSS) or Proportion.^50,51^ TSS is also called TC (Total count).^41,52,53^	Using the total read counts for each sample as the size factors to estimate the library size or scale the matrix counts Normalizing the count data involves dividing OTU (ASV/taxon/gene) read counts by the total number of reads in each sample to convert the counts to proportion. The total number of OTU (ASV/taxon/DNA fragments that are binned) in the sample is used to adjust the abundance of each OTU (ASV/taxon/gene).
Log transformation as normalization.	Log_2_ transformation is often used, which usually takes log_2_ (raw counts + a small pseudo value (e.g., 1)) transformation^54^ to make log(0) to be defined. Log_10_-normalization is defined as:^55^ $\log_{10}\left(\frac{RC}{n}\times \frac{\sum x}{N}+1\right)$, where RC represents the number of raw counts in a column OUT/ASV/gene table for a sample, n is the number of sequences in a sample, the sum of x is the total number of counts in the table, and N is the total number of samples.
**RNA-seq data-based normalization methods**	
Average genome size.^43^	Normalizing each metagenome by the average genome size. Averaging the genome size of the sampled organisms and the effects of genome size on the probability of sampling from a particular genome.
Quantile(Q).^50,56,57^	Transforming each sample to follow a data-derived reference distribution. Usually taking the median of all quantiles across the samples as the reference distribution.
Med (Median).^50^	Calculating the scaling factors based on the median OTU (ASV/taxon/gene) abundance. Calculating the normalization factor as the median of OTU (ASV/taxon/gene) counts that are nonzero in at least one sample.
Upper quartile (UQ).^50^ or log upper quartile (LUQ).^46^	Calculating the scaling (normalization) factors in a very similar way, in principle to TSS, except based on the upper quartile of the OTU (ASV/taxon/gene) abundances. Replacing the total counts by the upper quartile (75^th^ percentile) of OTU (ASV/taxon/gene) counts that are nonzero in at least one sample (Bullard et al. 2010). LUQ log transforming the counts after the scales each sample by the 75^th^ percentile of its count distribution. Using the upper quartiles of the OTU (ASV/taxon/gene) abundance distribution to calculate the scaling factors is to further increase the robustness in comparing to the median.
Conditional quantile normalization (CQN).^58^	Step 1: Setting a conditional Poisson statistical model to account for both correction for systematic biases and adjusting for distributional distortions. Step 2: Estimating the mean parameters of the conditional Poisson model to obtain normalized counts; that is, estimating the nonparametric functions along with the linear parameters that define the splines.
Smooth quantile normalization (qsmooth).^59^	Step 1: Calculating a reference distribution by averaging each quantile across the samples. each quantile across the samples. Step 2: Fitting a linear model with the known covariate for each quantile via modifying the functional normalization method.^60^ to relate the quantiles to a covariate that is associated with a biological group or condition. Step 3: Adaptively weighting the group information by estimating the regression coefficients representing the reference distributions within each biological group at each quantile and calculating the predicted values that correspond to the quantile normalized data within the biological groups. Step 4: Obtaining the smooth quantile normalized data by a weighted average of the observed quantile distribution for each sample.
Trimmed Mean by M-Values (edgeR-TMM).^61^	Calculating the scaling factors based on a robust analysis of the difference in relative abundance between samples. Assuming that most OTUs(ASVs/genes) are not differentially abundant and the abundances between the samples in average should be equal. Calculating the normalization factor using robust statistics: first, choosing a sample as reference; then, for each sample, filtering OTUs (ASVs/genes) based on their mean abundance and the fold- change between the sample and the reference; next, calculating the mean of the remaining log fold changes weighted by the inverse of the variance as an adjustment.
Relative Log Expression (DESeq-RLE).^61,62^	Calculating the scaling factors using the ratio between the OTU (ASV/gene) abundances and their geometric mean. Assuming most OTUs (ASVs/genes) are not differentially abundant. Calculating the normalization factor using the relative OTU (ASV/gene) abundances. First, creating a reference for each OTU (ASV/gene) by taking the geometric mean of its abundances across all samples. Then, calculating the normalization factor as the median of all ratios between the OTU (ASV/gene) counts in the sample and in the reference.
**Microbiome data-based normalization methods**	
**Mitigating over-dispersion normalization Methods**	
Cumulative sum scaling (metagenomeSeq-CSS).^42^	Calculating the scaling factors or the normalization factor for the sample as the cumulative sum of gene abundances up to a data-derived threshold. Assuming that the count distributions in each sample are equivalent for low abundant OTUs (ASVs/genes) up to a certain threshold, derived by the data. First, calculating the median of each lth quantile across all samples. Then, setting the threshold as the largest quantile. Setting the largest quantile leads to a sufficiently small difference between the sample-specific quantiles, which is measured based on the distance to the median quantile. By default, the thresholdshould be set to at least the 50^th^ percentile.
Reversed cumulative sum scaling (RCSS).^63^	Calculating the scaling factors as the cumulative sum of high abundant genes. As a variant of CSS, RCSS uses the observation that high-abundant genes in shotgun metagenomic data generally have a lower coefficient of variation. Calculating the normalization factor as the sum of all genes with an abundance larger than the median.
**Mitigating zero-inflation normalization Methods**	
Ratio Approach for Identifying Differential Abundance (RAIDA).^64^	Step 1: Adding a small number to the observed count and fitting the ratios of feature (or OTU) counts using a modified lognormal model. Step 2: Estimating the lognormally distributed mean and variance of ratios by the expectation maximization (EM) algorithm. Step 3: Selecting the possible common divisors. A set of clustered features common in both conditions is created based on the mean and variance of the ratios and its elements are as the possible common divisors. Step 4: Identifying non-differentially abundant features (DA-DAFs) or a group of non-DAFs as the common divisor through a moderated t-statistics^65^ for the log ratio of each feature using the estimated mean and variance.
Geometric Mean of Pairwise Ratios (GMPR).^66^	GMPR is based in RLE method, but reverses the order of the first two steps of RLE in calculating the size factor. That is, Step 1 is to calculate the geometric means for all OTUs and Step 2 is to calculate the median of the geometric means as the size factor for a given sample. Step 1 of GMPR is to calculate the median count ratio of nonzero counts between two different samples and Step 2 is to calculate the size factor for a given sample.
Wrench normalization.^67^	This method is a compositional bias correction technique for sparse count data, based on an empirical Bayes approach that borrows information across taxa and samples. Under the assumption that most taxa do not change across conditions, two steps are involved in deriving the sample-specific compositional correction/scale factors to estimate the systematic bias in a group and correct for compositional bias. Step 1: Using the averaged relative abundances (proportions) across the dataset as the reference. Step 2: Modeling the taxon-wise ratios of the observed relative abundances (proportions) as a hurdle log-normal model with the taxon-specific zero-generation probabilities, means, and variances.
**Compositionally aware normalization Methods**	
Centered Log-Ratio (clr) Transformation.^68^	clr is defined as the logarithm of the components after dividing by the geometric mean of x: $$Clr(x)=\left[In\left(\frac{x_{1}}{g_{m}(x)}\right),\ldots ,In\left(\frac{x_{i}}{g_{m}(x)}\right),\ldots ,In\left(\frac{x_{D}}{g_{m}(x)}\right)\right]$$ Where $x=(x_{1},\ldots ,x_{i},\ldots ,x_{D})$ represents the composition, and $g_{m}(x)=D\text{​}\text{​}\text{​}\text{​}\sqrt{x_{1}\cdot x_{2}\ldots x_{D}\cdot }$ is to ensure that the sum of the elements of clr(x) is zero. clr maps a composition in the D-part Aitchison simplex isometrically to a D-1 dimensional Euclidean vector.
Additive Log-Ratio (alr) Transformation.^69^	alr is defined as: $alr(x)=\left[In\left(\frac{x_{1}}{x_{D}}\right),\ldots ,In\left(\frac{x_{i}}{x_{D}}\right),\ldots ,In\left(\frac{x_{D-1}}{x_{D}}\right)\right]$ alr chooses one component as a reference and takes the logarithm of each measurement within a composition vector after dividing by a reference OTU (ASV/taxon/gene). alr maps a composition in the D-part Aitchison simplex non-isometrically to a D-1 dimensional Euclidean vector.
ANCOM-BC.^70^	ANCOM-BC normalization consists of three main steps: Step 1: Defining the sample-specific sampling fraction (consisting of the microbial load in a unit volume of the ecosystem and the library size of the corresponding sample) as the ratio of the expected observed abundance of taxon in a random sample to its unobserved absolute abundance in a unit volume of the ecosystem from which the sample was derived. Step 2: Estimating the sample-specific sampling fraction from the observed data. Step 3: Accounting for sampling fraction by including a sample-specific offset term to serve as the bias correction in a linear regression framework.
**Hybrid-based normalization methods**	
Network-and zero-inflated model-based normalization(RioNorm2).^71^	First, using the variance of the Aitchison’s log-ratio to measure the dissimilarity between two OTUs with respect to their co-abundance pattern across samples and conditions. Second, trimming the OTU table to keep the OTUs observed in at least 80% of the samples and the average count across all samples to larger than 5 readers. Third, calculatingthe pairwise dissimilarity using a network-based relatively invariant OTUs (riOTUs) searching algorithm. Fourth, building a network and finding a group of relatively invariant OTUs. Finally, calculating thesize factor as the sum of relatively invariant OTUs.
Cube root(cbrt)-based data normalization and normalization method aggregation.^72^	Step 1: for a data matrix with samples in rows and features/OTUs/ASVs in columns, using the cbrt normalization method to rescale data points in the dataset by first multiplying each of them by the standard deviation of the raw data, and then computing the cube root on the product to reduce the effect of the dominant features. Step 2: Combining the individual normalization methods^73^ including Cubic Root (CBRT) normalization, Minimum-Maximum normalization (MMN), Z-score normalization (ZSN), Variable Stability Scaling (VSS), and Pareto Scaling normalization (PSN) to extend the features of a dataset to produce a better method for addressing the feature dominance. Step 3: Combining data augmentation and feature extension to produce a more robust method that is used for augmentation or feature extension alone.

### Ecology data-based normalization methods

#### Rarefying as normalization

Rarefying plays the normalization role to adjust the sequencing depth in high-throughput sequencing research. The term rarefying originates in rarefaction.^49^ Rarefaction in physics refers to the reduction of an item’s density, as the opposite of compression, such as a form of wave decompression. Rarefaction method is a nonparametric resampling technique. In ecology, it was first proposed in 1968 by Howard Sanders to reduce the effect of sample size in diversity measurements.^45^

Rarefaction allows each sample to generate a line, so-called rarefaction curves, to calculate species richness for a given number of individual samples. These curves plot the number of species as a function of the number of samples. In general, rarefaction curves grow rapidly at first because the most common species are found and then plateau, as only the rarest species remain to be sampled. Thus, the rarefaction method has an advantage: it is dependent on the shape of the species abundance curve rather than the absolute number of specimens per sample.^45^ However, the individual-based taxon resampling curves have been evaluated as unstable performance:^40^ Depending on the settings, they could be justified for coverage analysis or species richness estimation,^74^ or could have worse performance than parametric methods.^75^

The idea of sampling, without replacement, in ecology’s rarefaction has been adopted by some microbiome researchers. This method has originally been commonly used to repeatedly assess alpha diversity (i.e., species richness) from the sampling effort^74,76^ and later has been used for the analysis of beta-diversity measures.^77,78^ Now, most major packages for the analysis of ecology and microbiome data, including mothur,^79^ QIIME,^80^ vegan,^81^ phyloseq,^48^ and microbiome,^82^ provide options of rarefying samples for normalization. However, to differentiate from the rarefaction in ecology, and to avoid confusion with rarefaction in physics and ecology, these researchers have intentionally used an alternative name, “rarefy” or “rarefying” or “rarefied counts”, when referring to the normalization procedure/results, instead of rarefaction. For instance, the term “rrarefy” has been used in QIIME,^80^ the term “rarefy” has been used in the phyloseq package,^48^ and the term “rarefy” in the microbiome package.^82^ Microbiome studies adopted the rarefaction method from ecology, but the rarefaction procedure is used in the sense that it randomly subsamples each sample to a common depth. In other words, it is used as an *ad hoc* procedure to normalize microbiome read counts that have resulted from libraries with widely-differing sizes, such as in QIIME.^80^

As a normalization step in microbiome studies, rarefaction method has been used not only for alpha diversity analysis, but also prior to beta diversity analysis.^77,83,84^ The rationale behind the subsampling depth choice is believed to be to strike a compromise between information loss and dataset balance. The researchers who use rarefaction method hope that decreasing the subsampling depth can improve a dataset’s balance, while it could also lead to the suboptimal use of the information contained in the dataset.^85^ ln microbiome literature, different thresholds of subsampling depth have been used: either the lowest number of sequences produced from any sample^77^ or even less,^84^ or arbitrary depth.^83^ This approach will remove any taxa or OTUs that are no longer present in any sample after random subsampling. The trade-off between number of samples and the depth of coverage has been evaluated; approximately 100 sequences per sample are generally sufficient to reveal the underlying gradient/beta diversity to obtain good qualities of clustering analysis, compared to the clustering observed when analyzing the complete dataset.^86^ These results demonstrate that increasing the number of sequences per sample does not necessarily improve the ability to detect ecological patterns.^86–88^

Aguirre de Cárcer et al.^85^ conducted a systematic evaluation of different subsampling depths and proposed a strategy for recoding singletons as zeros on the beta diversity measures. It showed that subsampling to the minimum as a normalization strategy did not perform particularly well, with data sets presenting some degree of coverage heterogeneity, while subsampling to the median did have benefit: it either improved the analysis or still retained a larger proportion of the initial sequences, when it had no effect. It also showed that multiple rarefaction with a recoding strategy, which randomly subsamples each sample to the median 100 times and then uses the average, and recodes values lower than 1.01 as zero, substantially improved the resolution of the analyses compared to both the initial and subsampling to the minimum strategies.^85^ However, McMurdie and Holmes (2014).^40^ showed that rarefied counts could result in an unacceptably high rate of false positive OTUs and failed to account for over-dispersion, resulting in a systematic bias that increases the Type-I error rate even after correcting for multiple-hypotheses. Thus, “rarefying microbiome data is inadmissible.”

Like in 16S rRNA-seq data, rarefying is also commonly used in shotgun metagenomics.^89,90^ For a review article, the reader is referred to.^91^ Rarefying can be implemented in phyloseq package.^48^ To correct for the different sequencing depth, the use of rarefying was reported in microbiome human breast cancer study,^92^ CRC study.^93–95^ and gut microbiome cancer and anti – PD-1 immunotherapy (Gopalakrishnan et al. 2018).^20^ We will discuss this topic further in Section 4.2.

### Traditional normalization methods

In the early development, like adopting rarefying for microbiome research, microbiome researchers adopted and most widely used scaling or size factor normalization methods to normalize both 16S rRNA and shotgun metagenomic microbiome data, including TSS^42,66^ or proportion,^40,46^ where normalization is based on scaling, first to estimate a sample-specific factor (size factor), and then using this sample-specific factor to correct the OTU(ASV/gene) abundances.

#### Total sum scaling (TSS) or proportion

In RNA-seq data, the proportion method was proposed in Bergemann and Wilson^96^ and evaluated by simulated data from exponential, Poisson, binomial, and normal models as well as real microarray and RNA-seq data. The proportion method averages the sum of the amount of mRNA in the test sample over the total amount of the expressed mRNA, represented by the sum of the test and reference samples.

For RNA-seq literature, TSS normalization has been evaluated to incorrectly bias differential abundance estimates^50,51^ because a few genes could be sampled preferentially as the sequencing yield increases in RNA-seq data derived through high-throughput technologies.

For 16S rRNA-seq data, TSS or proportion also has been reviewed to be having limitations, including 1) is not robust to outliers,^66^ is inefficient to address heteroscedasticity,^46^ is unable to address over-dispersion, and resulting in a high rate of false positives in differential abundance testing for species.^40,46,50,97^ This systematic bias increases the Type-I error rate even after correcting for multiple-testing.^40^ 2) TSS relies on the constant-sum constraint; hence it cannot remove compositionality and instead, is prone to create compositional effects, making nondifferential taxa or OTUs appear to be differential.^66,98–100^ Thus, due to strong compositional effects, TSS has a poor performance in terms of both false discovery rate (FDR) control and statistical power at the same false positive rate, compared to other scaling or size factor-based methods such as GMPR and RLE.^66^ 3) Especially, due to the nature of compositionality, proportions are criticized resulting in spurious correlations when comparing the abundance of specific OTUs relative to other OTUs.^101^

Actually, like rarefying, TSS or proportion is built on the assumption that the individual gene or taxon counts in each sample was randomly sampled from the reads in multiple samples. Thus, counts can be divided by the total library size to convert to proportion, and gene expression analysis or taxon abundance analysis can be fit via a Poisson distribution. Since in both RNA-seq data and 16S rRNA-seq data, the assumption cannot meet, the sample proportion approach is inappropriate for the detection of differentially abundant species,^40^ and should not be used for most statistical analyses.^46^ However, McKnight et al. (2019)^54^ demonstrated that although proportions are not suitable for differential abundance testing,^40,46,50,97^, proportion method is the most suitable method for community-level comparisons using dissimilarity and distance measures because the proportion method can produce the most accurate Bray-Curtis dissimilarities and the subsequent PCoA and PERMANOVA. Thus, they suggested that proportions (preferably) of rarefied data should be generally used for community-level comparisons.^54^

For shotgun metagenomic data, TSS or total count (TC) method has been evaluated having overall similar or higher performance than median and upper quartile,^91^ which is inconsistent with the evaluation results in RNA-seq data. Although theoretically, TC can be heavily dominated by the most common genes than the alternatives, median and upper quartile, because the alternatives replace the sum with the 50^th^ or the 75^th^ percentile of the gene count distribution as a scaling factor are more robust. However, Pereira et al.^91^ did not observe any tendencies that the median or the upper quartile had an overall higher performance than total count.

Total Sum Scaling (TSS) was reported from these microbiome CRC studies,^102^ and microbiome cancer diagnostic study.^103^ In another CRC study^104^ consensus reads were normalized by converting OTUs for each sample to a percentage of the reads for a given sample. The relative abundance (total-sum-scaled) data were also reported from the gut microbiome and cancer immunotherapy studies.^18,105^

#### Log-transformation as normalization

Generally, transformations, including log and power transformations, and variance stabilization normalization (VSN)^106,107^ have three overlapping goals^108:^ 1) correct for heteroscedasticity,^109^ 2) convert multiplicative relations into additive relations, and 3) make skewed distributions (more) symmetric. With the common belief that the log transformation can make data conform more closely to the normal distribution,^110,111^ it is expected to reduce the skewness of the data and the variability due to outliers. Thus, in practice, log transformation is used as a common remedy to deal with skewed data. It is used under the assumption that the log-transformed data have a distribution equal or close to the normal distribution.^111^

Log transformation has been reviewed having both benefits and drawbacks in metabolomics.^112^ For example, it is able to convert right-skewed data to be symmetric, to adjust heteroscedasticity, and to transform the relationship of metabolites from multiplication to addition,^108,113^ and particularly, it can perfectly remove heteroscedasticity for a relative constant standard deviation.^109^

However, log transformation: 1) cannot handle the zero value because log zero is undefined; 2) it has limited effect on values with a large relative standard deviation, which unfortunately is the usual case in microbiome data; and 3) it tends to inflate the variance of values near zero,^114^ although it can reduce the large variance of large values.

Log transformation has the general form of log (x, base), with the addition of the shift parameter to compute logarithms with any desired base. In microbiome study, the log_2_ transformation with adding a small pseudo value is commonly used. Log transformation with the addition of the shift parameter not only cannot help in reducing the variability, but also can be quite problematic to test the equality of means of two samples using log transformation when there are values close to 0 in the samples.

In microbiome literature, it was shown^54^ that log transformation suppresses large differences in common OTUs while amplifying slight differences in rare OTUs. In other words, log transformation tends to exaggerate the importance of differences among rare OTUs, while suppressing the importance of differences among common OTUs. Therefore, log transformation is generally not recommended for community-level comparisons because it distorts communities and alters species evenness. The log_2_-normalization method was used to normalize bacterium counts in a microbiome bladder cancer study^115^ and the log_10_-normalized counts were used in this microbiome CRC study.^8^

### RNA-Seq data-based normalization methods

Scaling or size factor normalization methods, most adopted directly from RNA-seq studies, first calculate the fixed values or proportions, called scale factors; then multiply the matrix counts by the scale factors, which is referred to as scaling the counts. The specific effects of the scaling methods are determined by the chosen scaling factors and how they are applied. RNA-seq data-based normalization methods commonly assume that the property of the majority of genes (in microbiome case, OTUs/ASVs/taxa) are not differentially abundant.

#### Quantile (Q)

Q method matches each lane’s distribution of gene read counts to a reference distribution, defined in terms of parameters quantiles such as simply scale counts within lanes by their median. In other words, similar to rarefying, Q normalization does not use scaling, instead it makes the gene abundance distributions in different samples identical by adjusting their quantiles based on a reference distribution derived by averaging over all the samples.^50,56,116^ The Q method was evaluated by RNA-seq data in.^50,97,117,118^ Previously, quantiles has been used to normalize single channel or A-value microarray intensities between arrays.^56,57^ In package limma, the quantiles method is used to normalize the columns of a matrix to have the same quantiles.^119^ Q normalization can be implemented in R package by adapting from the algorithm described in Bolstad et al.^56^ In practice, the median over the quantiles is typically calculated to preserve the discrete structure of the data, and one of the two middle values is randomly selected when the number of samples are even.^91^ Examples of Q normalization can be found in these CRC studies.^120^

#### Median (med)

In RNA-seq study, the median (Med) normalization method was proposed as a more robust alternative to the total counts (TC) method^50^ to normalize data from RNA-seq experiments. Med calculates the scaling (normalization) factors in a way similar in principle to TC: the total counts are replaced by the median (50^th^ percentile) of genes counts that are nonzero in at least one sample. Thus, the normalized data by Med is expected to be less influenced by most highly abundant genes. Median normalization can be performed via the edgeR package.^121^ Med has been evaluated by RNA-seq data in^50,51,61^ and application and evaluation in metagenomic data have been seen in^91,122^ Median normalization method was reported in microbiome CRC and immunotherapy studies.^123,124^

#### Log upper quartile (LUQ)

In RNA-seq studies, LUQ normalization method is typically called as upper quartile (UQ), which was first proposed and evaluated by Bullard et al.^50^ and also evaluated by Dillies et al.^51^ Overall, UQ, Med, DESeq-RLE and edgeR-TMM perform similarly on the varied data sets, both in terms of the qualitative characteristics of the normalized data and the results of the differential expression analysis. In microbiome literature, it is either referred to as the upper-quartile log-fold change (UQ-logFC)^40^ or log upper quartile (LUQ)^46^ to emphasize its application to log transformation. UQ normalization method estimates the scaling factors based on the 75^th^ percentile of the OTU(ASV/gene) count distribution. When the library composition across samples are different, such as the high-count OTUs(ASVs/genes) or a large number of zero counts present, then differences between the Med and UQ methods are expected.^51:^ Compared to the median method, the UQ normalization is expected to further increase the effects of robustness. In shotgun metagenomic data, UQ normalization method has been evaluated in.^91,122^ UQ (LUQ) normalization is implemented in the edgeR package.^121^ Examples of using the UQ normalization method are available from the microbiome lung cancer development study^125^ and the breast cancer tumor growth and metastatic progression study.^126^

#### Conditional quantile normalization (CQN)

CQN^58^ was proposed to remove the technical variability in RNA-seq data. The normalization algorithm is presented by a conditional Poisson statistical model to account for both the correction for systematic biases and adjusting for distributional distortions. It assumes that the log gene expression level for gene at a given sample is a random variable and the marginal distribution of the most log gene expression levels are independent and identically distributed across samples. In the conditional Poisson model, the mean parameter of the log gene expression level for the sample gene is modeled with three components: 1) A nondecreasing function to account for the nonlinear fact of count distributions across the different samples; 2) A nondecreasing function to account for sample-dependent systematic biases. These nondecreasing functions could be smooth functions that can be modeled as (parametric) natural cubic splines; and 3) a log parameter to adjust for the sequencing depth in millions. The use of CQN has two advantages: First, compared to scaling-based normalization methods.^50,61,127^ such as edgeR-TMM that only can provide more robust estimates of the shift in location, CQN has the advantage of resulting in sample distributions with comparable scales and shapes.^58^ Since RNA-seq data exist with several sources of unwanted technical variability, such as the differences in cDNA amplification efficiency and other technical artifacts as well as the differences in distribution shapes and scales that persist after accounting for library size, a normalization based on robust estimates of both scales and shapes is important. Second, compared to RPKM normalization,^128^ its performance shows a strong dependence on fold change and guanine-cytosine content (GC-content). CQN has shown substantial improvement and eliminated the dependence on fold change and GC content. The reason is that RPKM normalization takes gene length into account to normalize genes by dividing by the gene length, and assumes that the effect is considered static and constant for all samples. However, CQN has the disadvantage: it needs a large amount of data for each sample and a parsimonious model to define a stable algorithm. The CQN method can be implemented via the R Bioconductor package cqn. CQN was reported from breast cancer microbiota and host gene expression study to normalize the host gene expression counts.^129^

#### Smooth quantile normalization(qsmooth)

qsmooth^59^ was proposed based on the assumption that each sample should have the same statistical distribution (i.e., have the same shape of distribution) within biological groups (or conditions), but statistical distributions of the samples between the groups may differ. qsmooth takes the approach of normalization in terms of global transformations, generalizing quantile normalization. However, different from other global normalization methods, which assume that the observed variabilities in global properties (e.g., differences in the total, upper quartile or median gene expression, and proportion of differentially expressed genes) are caused by technical reasons and are unrelated to the biology of interest. qsmooth aims to remove both systematic bias and unwanted technical variation in high-throughput data.

qsmooth is a generalized quantile normalization, in which a weight is computed for each quantile by comparing the variability between groups, relative to the total variability between and within groups. The weight is based on the variability between groups. The less variability between groups, the larger the weight toward one, and the quantile is shrunk toward the overall reference quantile. Thus, depending on the biological variability, the weight may vary between 0 and 1 across the quantiles. In one extreme case, when there are no global differences in distributions, i.e., global differences in distributions correspond to differences in biological groups, qsmooth is similar to standard quantile normalization within each biological group, then the weight is zero.

It was demonstrated^59^ that qsmooth has advantages including

1) preserving global differences in distributions, corresponding to different biological groups, while scaling normalization methods, e.g., UQ, edgeR-TMM, DESeq-RLE (see below) could not well control the variability between distributions within tissues; 2) reducing the root mean squared error (RMSE) of the overall variability across distributions, and particularly, compared to Q normalization, qsmooth normalization preserves the tissue-specificity, whereas Q normalization removes the biologically known tissue-specific expression; and 3) maintaining a better bias-variance tradeoff with an increasing small bias but a tradeoff with a reduction in variance.

However, qsmooth adds pseudo count of 1 to all counts and then performs log 2 transformation of the counts. This transformation approach has a limitation. qsmooth can be implemented via the R package qsmooth. qsmooth was used by combining with the batch effect correction method to remove known batch effects using a bladder cancer gene expression data.^59^ Another example of using qsmooth in cancer study is available from the colon cancer drug metabolism study.^130^

#### Trimmed mean of M-values (edgeR-TMM)

TMM method was proposed based on the hypothesis that most genes are not differential expressed(DE).^121^ Applying TMM to 16S rRNA-seq and shotgun metagenomic data, the TMM factor is calculated for each sample, with one sample set as a reference sample and the others as test samples to compare the OTU (ASV/taxon/gene) abundances in the samples. For each test sample, the TMM scaling factor is calculated as the weighted trimmed mean of log ratios between each pair of this test and the reference, after excluding the highest OTUs (ASVs/taxa/genes) and the OTUs (ASVs/taxa/genes) with the largest log ratios or log-fold change. By default, 30% of the log fold change and 5% of the mean abundance are trimmed. This minimizes the log fold change between the samples for most OTUs (ASVs/taxa/genes).^61,121^ Due to the assumption that the majority of OTUs (ASVs/taxa/genes) are not differentially abundant, like the scaling factors in DESeq, the TMM scaling factors are usually around 1. However, different from DESeq, its scaling factors applies to read counts; TMM applies scaling factors to library sizes. The performance of edgeR-TMM has been evaluated by RNA-seq data in,^131^ by 16S rRNA-seq data in,^40,46,54,66^ and by shotgun metagenomic data.^91^ The edgeR-TMM normalization is implemented in the edgeR package,^121^ which by default, trims 30% of the log fold change and 5% of the mean abundance. The examples of using edgeR-TMM normalization method are from the CRC study^8^ and the lung cancer study.^132^

#### Relative log expression (DESeq-RLE)

DESeq normalization method is included in the DESeq package, which was proposed based on the hypothesis that most genes are not differential expressed (DE). DESeq calculates the scaling factor for a given sample as the median of the ratio for each gene, its read count over its geometric mean across all samples.^127^ The calcNormFactors() function in the edgeR package^121^ can calculate RLE normalization factors when the setting method = “RLE” and the TMM normalization factors when the setting method = “TMM”. To avoid confusion, these two normalization methods are sometimes referred to as the edgeR-RLE normalization,^40^ and edgeR-TMM normalization,^46^ respectively.

The default normalization methods used in edgeR and DESeq are similar. Ander and Huber^127^ thought that their DESeq normalization method is similar to the relative log expression normalization method (“RLE”), implemented in edgeR and proposed by Robinson and Oshlack.^61^ To avoid confusion, we call DESeq normalization method as DESeq-RLE. The edgeR and DESeq normalization methods have similar purposes:^133^ 1) both use normalization to adjust for sequencing depths because different depths are sequenced by different libraries, and 2) both use the normalizations as internal procedure, that is, are built in the statistical model as offsets to often ensure that parameters are comparable, while without actually altering the raw read counts.^61^ However, the default normalization methods in the packages DESeq and edgeR, are different because “edgeR uses the trimmed mean of *M* values, whereas DESeq uses a relative log expression approach by creating a virtual library that every sample is compared against”, although in practice, “the normalization factors are often similar”.^134^ Thus, we do not confuse the DESeq-RLE with edgeR-RLE (relative log expression normalization). Actually, the results obtained from the functions calcNormFactors() with method = “RLE” in the edgeR package (edgeR-RLE) and estimateSizeFactorsForMatrix () in the DESeq(2) package are different. Because the function calcNormFactors() works with pre-normalized counts rather than with raw counts,^135^ RLE in the edgeR package needs to be further normalized to be identical to the DESeq method and to present as DESeq-RLE.^136^ The following quote from the authors of DESeq can best describe the differences and similarities between these two normalization methods.^134:^

By default, edgeR uses the number of mapped reads (i.e., count table column sums) and estimates an additional normalization factor to account for sample-specific effects (e.g., diversity);^61^ these two factors are combined and used as an offset in the NB model. Analogously, DESeq defines a virtual reference sample by taking the median of each gene’s values across samples and then computes size factors as the median of ratios of each sample to the reference sample. Generally, the ratios of the size factors should roughly match the ratios of the library sizes. Dividing each column of the count table by the corresponding size factor yields normalized count values, which can be scaled to give a counts per million interpretation. (see also edgeR’s *cpm* function)

To estimate these size factors, the second version of DESeq package (DESeq2) offers the same normalization method that is already used in DESeq, called “the median-of-ratios method”.^62^ But DESeq2 has the advantage to calculate gene-specific normalization factors to account for further sources of technical biases, such as differing dependence on GC content, gene length or the like.^62^ Thus, in this review article, we name the “Relative Log Expression” normalization that is implemented by default in the DESeq and DESeq2 packages as DESeq-RLE, which is considered equivalent to the DESeq-median-of-ratios method (label as the “median-of-ratios method”). We name the relative log expression normalization (RLE) that implemented in edgeR^121,127^ as edgeR-RLE to differentiate it from DESeq-RLE.

Like edgeR-TMM, the effective application of DESeq-RLE to 16S rRNA-seq data need to assume most taxa or OTUs are not differentially abundant. Then scaling factors can be calculated by comparing the samples to a reference. However, different from TMM setting one sample in the study as a reference, DESeq-RLE instead uses a pseudo-reference which is calculated for each taxon or OTU by taking the geometric mean of its abundances across all samples. The normalization factors are then calculated as the median of all the ratios between taxon or OTU counts between samples and the reference. Choosing the median is to prevent the large count values of taxa or OTUs from having undue influence on the values of other taxa or OTUs. Then, using the scaled counts for all the taxa or OTUs and assuming a Negative Binomial (NB) distribution, a mean-variance relation is fit. DESeq-RLE through using a log-like transformation in the NB generalized linear model (GLM) to adjust the matrix counts, with expect to that the variance in the taxon or OTU’s counts across samples is approximately independent of its mean.^127^

Although edgeR-TMM and DESeq-RLE normalization methods are slightly different, one common characteristic of them is that both are used in NB model along with offset to stabilize variance. Thus, DESeq normalization method has been referred to as relevant variance stabilization transformation (DESeqVS).^40,46^ In summary, the development of normalization methods that were adopted directly from RNA-seq study have two characteristics: 1) Using a stable and robust value such as median or 75% percentile of count as reference; and 2) Using NB model to address over-dispersion.

In RNA-seq data, it was evaluated^51,131^ only DESeq-RLE and edgeR-TMM are able to control the false-positive rate while also maintaining the power to detect differentially expressed genes, especially in the presence of high-count genes. In 16S rRNA-seq data, different evaluation conclusions were achieved: on one hand, it was shown^40^ that DESeq-RLE and edgeR-TMM had better performance than rarefying and proportion methods based on differential abundance analysis. On the other hand, it was shown^46,54^ that DESeq-RLE and edgeR-TMM had worse performance in community-level comparisons compared to rarefying and proportion methods. The edgeR-TMM and DESeq-RLE methods were also evaluated having better performance by shotgun metagenomic data,^91^ which is consistent with previous evaluations by RNA-seq data^51^ and 16S rRNA-seq count data.^40^ DESeq-RLE normalization can be performed by calling the estimateSizeFactors() and sizeFactors() functions in the packages DESeq^127^ and DESeq2.^62^ DESeq-RLE was reported from the microbiome prostate cancer study,^17^ and microbiome GI tract cancer study^2^, and a microbiome breast cancer study.^137^

#### Other library size/subsampling-based normalization methods in RNA-seq data

Other RNA-seq data normalization methods were also adopted into microbiome cancer research.

To remove the data’s heteroskedasticity and to not perform imputation of missing sequences, sometime unaltered reads per kilobase of transcript per million mapped reads (RPKM) or fragments per kilobase of transcript per million mapped reads (FPKM) or counts per million (CPM) or log-count per million (log-CPM) data normalization method via the Voom algorithm^138^ is used. For example, the count abundance of oral microbiota across samples was normalized to one million counts in an oral microbiome cancer study.^139^ The gene abundance table was normalized by the FPKM strategy, in which the gene abundance is normalized by the gene size and the number of total mapped reads reported in frequency.^20^ In RNA-seq and micorbiome literatures, the “reads per kilobase per million” (RPKM)^128^ normalization was often used.^18,105,140^

#### Microbiome Data-Based Normalization Methods

We categorize the microbiome data-based normalization methods into four groups: 1) toward mitigating over-dispersion, 2) toward mitigating zero-inflation, 3) toward mitigating compositionality, and 4) hybrid-based normalization methods.

#### Normalization methods toward mitigating over-dispersion

##### Cumulative sum scaling (metagenomeSeq-CSS)

CSS normalization^42^ is a normalization method, specifically designed for microbiome data and is implemented via metagenomeSeq package. CSS aims to correct the bias in the assessment of differential abundance, introduced by total-sum normalization. CSS method is to divide raw counts into the cumulative sum of counts, up to a percentile, determined using a data-driven approach so as to capture the relatively invariant count distribution for a data set. In other words, the choices of percentiles are driven by empirical rather than theoretical considerations.

CSS method is an adaptive extension of the quantile normalization method.^50^ CSS is a bridge of normalization methods of RNA-seq and 16s rRNA-seq data. It adopts both strategies used in RNA-seq study; that is, combining a stable and robust value (by default, 50^th^ percentile is set as the threshold) and a NB model to mitigate over-dispersion of microbiome data. MetagenomeSeq uses a log transformation (log_2_(y_ij_+1)) followed by correction for zero-inflation, based on a Gaussian mixture model,^42^ and performs a statistical inference after transformation, using a normal inverse gamma empirical Bayes model to moderate the gene-specific variance estimates.^141^ Thus MetagenomeSeq functions to address over-dispersion and zero-inflation. Although the CSS method is coupled with a zero-inflated model in metagenomes package, aiming to handle the high number of zero observations encountered in the metagenomic data, the CSS method itself is a normalization method for mitigating over-dispersion of the microbiome data.

In summary, CSS normalization was developed to address the influence of larger microbiome count values. In 16S rRNA data, this method has been evaluated to having several advantages, including: 1) CSS is similar to LUQ, but more flexible than LUQ because it allows a dependent threshold to determine each sample’s quantile divisor.^46^ Particularly, it showed that 2) CSS, along with GMPR, is overall more robust to sample-specific outlier OTUs than TSS and RNA-seq normalization methods;^66^ 3) CSS has a higher performance compared to similar algorithms on sparse data.^67,142;^ and 4) CSS may have superior performance for weighted metrics, although it is arguable.^42,46,143,144^ In shotgun metagenomic data, Pereira et al.^91^ also showed that CSS, along with edgeR-TMM and DESeq-RLE, has a high overall performance for larger group sizes and particularly has a good performance at controlling the FDR, even for highly unbalanced effect. The evaluation results are in line with the overall finding in 16S rRNA-seq that metagenomeSeq CSS performs well when there is an adequate number of biological replicates.^40^ The high overall performance for larger group sizes may be because CSS optimizes, including genes, to calculate the scaling factor, and hence minimizes the variability. However, CSS has the challenge to determine the percentiles for microbiome data sets with high variable counts^66^ and has been particularly evaluated to yield a large number of false positives even though metagenomeSeq has a high power with small effect size,^71^ having among the worst performance for the low group size.^91^ Thus, Pereira et al.^91^ strongly suggested that CSS normalization method should only be applied to data sets with sufficiently many samples. CSS normalization is implemented in the metagenomeSeq package.^145^ Please note that in the most recent version (version 1.34.0), metagenomeSeq prefers to use the wrench normalization (WN) method over CSS. CSS algorithm was used in the CRC study to normalize both gene frequency data and taxonomic data.^146,147^

###### Reversed cumulative sum scaling (RCSS)

RCSS.^63^ was developed as a variant of CSS in shotgun metagenomic data in which the normalization factor is calculated as the sum of all genes with an abundance larger than the median. Like TC, UQ and Q normalization methods, RCSS was evaluated producing highly skewed *P*-value distribution and hence resulting in biased FDRs when the effect of differentially abundant genes becomes unbalanced between the groups.^91^ RCSS can be implemented in R using the function colQuantiles() from the matrixStats package and the function sum() over a logical vector. RCSS is included in MetaAnalyst^148^ as one of five normalization methods (i.e., total counts, median, upper quartile, reversed RCSS, and z-score) of data preprocessing for subsequent analysis.

#### Normalization methods toward mitigating zero-inflation

##### Ratio approach for identifying differential abundance (RAIDA)

RAIDA^64^ was developed using the ratio between features (OTUs/taxa) in a modified zero-inflated lognormal model to identify differentially abundant features (DA-DAFs) or differentially abundant OTUs (DA-OTUs). As a normalization method, the development of RAIDA was motivated by the undesirable results produced when applying the total sum, mean, or median normalization methods to normalize the large amount of differences in total abundances of DA-DAFs across different conditions. Like edgeR and DESeq, RAIDA assumes that the majority of features (or OTUs) are not differentially abundant, and uses the ratio between the counts of features (or OTUs) in each sample to eliminate possible problems associated with counts on different scales, within and between conditions. Because microbiome data have many zeros and the ratio of zeros is undefined, RAIDA assumes that most zero values are due to undersampling^149^ of the microbial community or of insufficient sequencing depth. Thus, RAIDA uses a modified zero-inflated log-normal model to calculate size factors by adding a small number to the observed count of each feature (or OTU) for each sample before computing the ratios. A lognormal distribution is often assumed for non-zero ratios in the compositional data analysis.^69^ By adding a small number to the observed count, the original ratios of zeros can be fit with a modified lognormal model.

Sohn et al.^64^ showed that the performance of RAIDA 1) is not affected by the total different abundances of DA-DAFs (DA-OTUs) across different conditions and hence, RAIDA can remove possible problems associated with counts on different scales, within and between conditions; and 2) compared to edgeR-TMM,^121^ Metastats,^41^ and metagenomeSeq^42^ normalization methods, RAIDA performs consistently, powerful for both the balanced and unbalanced conditions, and hence greatly outperforms these existing methods. For example, metagenomeSeq and RAIDA can provide better power to detect the ratio of true positives to positives, but metagenomeSeq could significantly increase FDR in both the balanced and unbalanced conditions. The edgeR-TMM performs best for the balanced conditions in terms of controlling FDR, However, for the unbalanced conditions, as the percent of DAFs increases, RAIDA surpasses edgeR. Another simulation study^66^ demonstrated that DESeq2 and RAIDA are overall more robust and powerful than edgeR and metagenomeSeq, and are able to control the FDR, close to the nominal level. In summary, RAIDA is effective at controlling FDR close to the nominal level of 0.05, regardless of the outlier, which confirms that the method is robust. Like DESeq(2) is accompanied with a NB model, RAIDA is accompanied with a moderated *t*-statistics, which was evaluated as having very low power in detecting differential abundance for small or medium effect sizes and its overall performance is highly sensitive to the library size.^71^ For example, when effect sizes and library sizes are small, RAIDA underperforms compared to RioNorm2.^71^ RAIDA was reported to having been used to identify the differentially abundant gut microbes between CRC and healthy samples.^150^

##### Geometric mean of pairwise ratios (GMPR)

GMPR^66^ was specifically proposed for zero-inflated sequencing data such as microbiome sequencing data. As we described above, compared to RNA-Seq data, microbiome sequencing data are more severely over-dispersed and zero-inflated. However, the existing normalization methods, including the traditional and the RNA-seq data-based normalization methods as well as CSS normalization, which was specifically designed for microbiome data, cannot effectively handle over-dispersed and zero-inflated microbiome data. Motivated by the inability of these available normalization methods to normalize microbiome sequencing data, GMPR was particularly developed to normalize zero-inflated count data, with application to microbiome sequencing data and to other sequencing data with excess zeros, such as, single-cell RNA-Seq data.^66^

GMPR normalization method extends the idea of RLE normalization for RNA-seq data and relies on the same assumption that the large part of count data is invariant in the 16S OTU-count table. The reason that GMPR reverses the order of the first two steps of RLE method is that RLE fails for OTUs with zero values because geometric mean is not well defined for zero. It was demonstrated^66^ that GMPR outperforms RNA-Seq and CSS normalization methods mainly because it 1) is robust to differential and outlier OTUs, 2) improves the performance of differential abundance analysis and the reproducibility of normalized abundance, and 3) reduces the inter sample variability of normalized abundances. However, GMPR method also has some limitations.^66:^

1. Its appropriateness relies on the assumption that most taxa or OTUs in the count data are invariant.^66^ However, as in RNA-seq data, actually in practice, the assumption may not meet in some 16S rRNA-seq data and the assumption would even be extremely difficult to check,and have been rarely checked.^51,151^
2. It is mainly applied to taxon-level analysis of differential abundance and its reproducibility could be improved to identify the “truly” differential taxa under the compositional context.
3. It is computationally complicated and inefficient for large samples sizes.
4. GMPR is accompanied by an omnibus test using a zero-inflated negative binomial (ZINB) distribution, which was evaluated as having very low power in detecting differential abundance for small or medium effect sizes, and its overall performance is highly sensitive to the library size.^71^
5. It was shown^71^ that if the study is interested in the absolute abundance counts instead of the relative abundance (i.e., proportion) and when the effect size increases, then it is inappropriate to use the omnibus test because, like DESeq, DESeq2, and metagenomeSeq, it tends to detect more false positives. GMPR is implemented with the GMPR R package. GMPR were reported in an association study of cancer and microbiome^152^ and in a study comparing the fecal microbiome of CRC patients and healthy controls as a function of age.^153^

##### Wrench normalization (WN)

Wrench normalization^67^ was proposed to analyze and correct compositional bias in sparse sequencing count data. Here, compositional bias refers to the bias that occur inherently due to the sequencing process, which produce the taxa abundances to be relative (compositional) rather than absolute. Without the correction of the compositional bias, inference of absolute abundances will be confounded. The WN method belongs to the count-based data normalization approach, which is different from the approach that uses log-ratio family to address the compositionality. We will review the log-ratio-based approach in Section 3.4.3. The WN method was developed based on two assumptions.^67:^ 1) most taxa do not change across conditions/groups; and 2) zero observations in samples that are mainly caused by sequencing technology are correlated with compositional changes. The WN method is used along with a count-based hurdle log-normal distribution model, in which the probabilities of zero values are determined by sample covariates, including the total sequencing depth, and the positive count value is modeled as a log-Gaussian random variable. The mean of log-Gaussian random variable is determined by 1) the chosen log-reference value, 2) log-sample-depth, 3) the log-net fold change relative to reference (the log of sum of group-wise effect, two-way group-sample interaction, a three-way group-sample-taxon interaction random effect), and 4) a noise term.

WN was developed with these backgrounds: On one hand, in library size/subsampling-based approaches (e.g., TC, RPKM, FPKM, CPM), the normalization factors are adjusted by sample depths, but usually the taxon high intra- and inter-group taxon diversity is not adjusted in their general experimental settings via the framework of generalized linear models. On the other hand, the reference-based normalization and robust fold-change estimation approaches such as edgeR-TMM and DESeq methods overcome compositional bias at high sample depths, significantly take account for the taxon high intra- and inter-group taxon diversity, and hence outperform library size-based approaches. However, such techniques were originally developed for bulk RNA-seq data and face major difficulties with sparse 16S rRNA count data.^67^

Different from existing normalization approaches, WN derives the estimate of compositional correction factor for every sample, based on the ratio of its proportions to that of the reference. The compositional correction factors are estimated to 1) adjust for the taxon-wise values within and across samples, 2) smooth the taxon-wise estimates across samples before deriving the sample-wise factors using an empirical Bayes strategy, which makes the computed factors robust to low sequencing depths and low abundant taxa.

In summary, WN is a reference-based compositional correction method and can be broadly viewed as a generalization of edgeR-TMM^61^ for zero-inflated data.

However, unlike existing commonly used count-based data normalization techniques, in that, either TMM/DESeq/CSS normalization methods results in loss of information due to ignoring zero values or library size scaling (e.g., unaltered RPKM/CPM), rarefaction/subsampling can only correct the technical bias that is correlated with library size but cannot correct for compositional bias due to sparsity in 16S rRNA-seq count data.^67^ It was shown^67^ that the WN method has a better normalization accuracy, leading to reduced false positive calls in differential abundance analysis and improved quality of positive associations to reconstruct precise group-wise estimates and provide rich annotation in discoveries. Especially, it can still offer robust protection against compositional bias for low sequencing depths and low abundant taxa at higher coverages. Thus, it is a better alternative for under-sampling microbiome data.

Overall, the WN method, paired with the zero-inflated log-normal regression, tends to be more accurate and robust to sparsity. Thus, this method has better performance in terms of controlling the FDR while maintaining the higher power. The advantage in this approach may lie in considering zeros, adjusting both the intra- taxon and inter-group taxon variabilities, and incorporating group information to provide more reliable estimates. The benefits of using WN method, paired with the zero-inflated log-normal regression, to analyze sparse 16S rRNA microbiome data have been evaluated in.^154,155^ However, since this method assumes that most taxa do not change across conditions/study groups, on average, and uses the average of the ratios of relative abundances across taxa as the estimated scaling factor, the WN method would still suffer in the analysis of taxa arising from arbitrary general conditions.^67;^ In other words, it faces challenges when the effect sizes of differentially abundant taxa are too large.^156^ Additionally, the current version of Wrench approach is not flexible; it only supports two-group comparison and cannot adjust for continuous covariates (e.g., age, time) or cannot normalize data based on continuous covariates.^67^ A Bioconductor R package Wrench is available for Wrench normalization for sparse count data.^157^

#### Normalization methods toward mitigating compositionality

A data is defined as compositional if it contains multiple parts of nonnegative numbers, whose sum is 1^69^ (p.25) or any constant-sum constraint^158^ (p.10). That is, compositional data can be represented by a constant sum of real vectors with positive components, such as per unit (proportions), percent, parts per million, and parts per billion. Because compositional components are dependent, which violates the assumption of independence, when applied to the standard statistical methods, usually compositional data are transformed prior to statistical analysis. Compositionally aware transformation can be considered as normalization. Several such transformations have been proposed in literature, including the center log-ratio transform (clr),^68,159^ additive log-ratio transform (alr),^69,159^ isometric log-ratio transform (ilr),^160^ the inter quartile log-ratio (iqlr),^161,162^ and sequential binary partitions or balance trees,^163,164^ which uses phylogenetic trees data to guide the ilr transformations. Among them, the transforms clr, alr, and iqlr are adopted into ALDEx2 (clr-and iqlr-based method),^162^ and ANCOM(alr -based method),^98^ the two most often used microbiome compositional software, to transform/normalize microbiome data to account for the compositional structure of microbiome data.

**clr-transformation and ALDEx2**

It was shown^162^ that the clr-transformation algorithm could be used to analyze next-generation sequencing data, including RNA-seq and microbiome data, and it has been adopted into some software, including ALDEx2(ALDEx).^161,165^ ALDEx2 was developed to find the differential expression of genes, relative to the geometric mean abundance between two or more groups. There are two benefits of using clr-transformation: 1) It is able to remove the unit-sum constraint of compositional data, hence allowing ratios of components to be analyzed in the Euclidean space; and 2) taking the ratio with respect to the geometric mean of the whole composition leads data to fall into a *P*-1 hyperplane of *P*-dimensional Euclidean space, while after the transformation, the data remains in *P*-dimension. Furthermore, ALDEx2 has been demonstrated as having these advantages: 1) is robust.^161;^ 2) has very high precision in identifying differentially expressed genes (and transcripts) for 16S rRNA data and has high recall, given sufficient sample sizes;^166^ and 3) has the potential to be generalized for use in any type of high-throughput sequencing data.^167^ However, ALDEx2 has several drawbacks, including: 1) It uses a value from valid Dirichlet distribution to replace zero read counts,^161^ thus it cannot address the zero-inflation problem. 2) The performance of ALDEx2 statistical testing is dependent on transformations; when the log-ratio transformation cannot sufficiently approximate an unchanged reference, it is difficult to interpret the testing results of ALDEx2.^168^ At this point, the inter-quartile range log-ratio (iqlr) transformation outperforms the centered log-ratio (clr) transformation, and was recommended for use as the default setting for ALDEx2.^166^ 3) Because ALDEx2 performs statistical hypothesis testing using nonparametric methods (Wilcoxon rank sum test or Kruskal-Wallis test for comparisons of two or more than two groups, respectively), it reduces statistical power and hence requires large sample sizes.^166,168–171^ For example, it was reported that ALDEx2 is difficult to control FDR^156^ and to maintain statistical power compared to competing differential abundance methods (e.g., ANCOM, ANCOM-BC, edgeR, and DESeq2).^156,172^ Centered log-ratio transformation was used in the gut microbiome and cancer immunotherapy studies^105,173^ to account for the compositional nature of microbial sequencing data.

**alr- transformation and ANCOM**

ANCOM^98^ is an alr (additive log-ratio)-based method because it was developed based on alr- transformation, to account for the compositional structure of microbiome data. ANCOM repeatedly uses alr-transformation to choose each of the taxa in the data set as a reference taxon, at a time, and builds its statistical tests on point estimates of transformed OTU counts. After the alr- transformation, the different ratios revealed between the groups can be analyzed by standard statistical tools.^174^ The use of ANCOM has these benefits: 1) It can well control the FDR while maintaining power comparable to other methods.^70,175^ 2) Especially, compared to RioNorm2 and RAIDA, it has very low FDR (close to 0) and hence is able to detect differential abundant OTUs.^71^

However, like ALDEx2, in ANCOM, log-ratio transformations play a role in normalization (log-ratio ‘normalizations’). Thus, ANCOM suffers from some similar drawbacks as ALDEx2: 1) Its appropriateness majorly depends on whether the log-ratio transformation sufficiently approximates an unchanged reference.^168^ The compositional transformed data when using alr, clr, and ilr transformations not only do not improve raw counts tables, but also often make the performance of statistical analysis worse. In other words, the methods that do not require using log-ratio ‘normalizations’ are more appropriate.^168^ 2) Compositional analysis approach cannot address zero-inflation problem; rather it fails in the presence of zero values.^176^ (p.389). ANCOM could increase the rate of false positives rather than control the FDR due to its improper handling of the zero counts.^177^ 3) The nonparametric nature of compositional analysis approach requires a large number of samples and is underpowered^166,168^ as well as it decreases sensitivity on small data sets (e.g., less than 20 samples per group).^46,168^ Additionally, ANCOM has the following drawbacks: 1) It is computationally intensive to repeatedly apply alr transformation to each taxon in the data set as a reference taxon and it is also a challenge in choosing the reference taxon when the data set has larger number of taxa. 2) No consistent conclusions have been achieved regarding whether ANCOM can control FDR well or not: some studies reported that ANCOM can control FDR reasonably well under various scenarios,^46,70^ whereas others showed that ANCOM could generate a potentially false-positive result.^172^ 3) ANCOM performs statistical testing for significance using the quantile of its test statistic, W, rather than the *P*-values, which not only results in the analysis results difficult to interpret,^156^ but also does not improve the performance of ANCOM by filtering taxa before analysis. ANCOM was reported to have reduced the number of detected differential abundant taxa. This is most likely related to the way that W statistics are calculated and used for significance in ANCOM.^178^ 4) ANCOM does not provide the *P*-value for individual taxon and cannot provide standard errors or confidence intervals of differential abundance analysis for each taxon.^70^ 5) The differential abundance testing results are difficult to interpret because ANCOM uses presumed invariant features to guide the log-ratio transformation.^168^ Examples of microbiome cancer studies using ANCOM are available from these reports.^179–181^

##### ANCOM-BC

ANCOM-BC^70^ was proposed for differential abundance analysis (DDA) of microbiome data, with bias correction to ANCOM. ANCOM-BC assumes that 1) the observed taxon abundance is proportional to the unobservable absolute taxon abundance in a unit ecosystem; and 2) the estimation bias is caused by the sampling fraction of variation. ANCOM-BC is a two-stage method that begins with a normalization step and followed by the linear model framework for log-transformed OTU counts data. The goal of ANCOM-BC normalization is to eliminate the differential sampling fractions between each group and allow inferencing the absolute abundance from relative abundance. To address the problem of unequal sampling fractions, it uses a sample-specific offset term to serve as the bias correction.^70^ The offset term is usually used in the generalized linear models to normalize or adjust for the library size across samples.^182^ Here,ANCOM-BC uses a sample-specific offset term in a linear regression framework. However, here the sample-specific sampling fractions include both the library size of the corresponding sample and the microbial load in a unit volume of the ecosystem. Thus, in ANCOM-BC, both the library size across samples and the differences in the microbial loads are normalized or adjusted for.

ANCOM-BC has some advantages,^70^ including: 1) More sample biases are corrected than other library size adjustment approaches. 2) It can well control the FDR while maintaining adequate power, compared to other count-based methods such as edgeR and DESeq2. In RNA-seq studies, the zero-inflated Gaussian mixture model is used in metagenomeSeq.

However, ANCOM-BC also has some weaknesses: 1) It cannot well control the FDR for sample sizes less than 5 per group.^70^ 2) It assumes that the compositional data on the covariates is linear, which is not often true and it cannot control the FDR well when this linear assumption is violated.^183^ 3) In practice, it uses the pseudo-count approach to impute the zeros and excludes those taxa that are associated with structural zeros in the analysis,^184^ resulting in decreased performance. 4) It has a better FDR control than TSS-based methods. However, it still has low performance under strong compositional effects with Type I error inflation or low statistical power.^155^ Please note that although ANCOM-BC attempts to provide further scale adjustments for compositionality and sparsity, it uses log- transformation of OTU counts data rather than log-ratio transformation. Thus, its methodology is not a typical compositional data analysis in the sense of Aitchison’s approach.^69^ To solve the problem of compositionality, it uses the sum of ecosystem as reference to offset the data. This kind of normalization is not the typical Aitchison’s approach-based normalization. ANCOM-BC was reported to compare with other methods, in association with human microbiome, in the colorectal cancer study.^185^

#### Hybrid-based normalization methods

In microbiome literature, some kinds of hybrid normalization methods have been developed in terms of explicitly combining or aggregating normalization procedures and statistical analysis modeling.

##### Network- and zero-inflated model-based normalization(RioNorm2)

The scaling or size factor normalization methods are developed under the assumption that the sampling counts are equivalently distributed up to a certain percentile, and hence the counts on different scales can be adjusted to a common scale using size factors. However, one common limitation of this kind of methods is that it may be ineffective to detect low to medium abundance level of taxa because they could result in false positives, and have lower power to detect taxa with small effect size.

RioNorm2^71^ was developed along with a two-stage zero-inflated mixture count regression model, aiming to find a group of relatively invariant microbiome species across samples and conditions to construct the size factor for normalization, where the normalized count data is analyzed by a two-stage zero-inflated mixture count regression model: First, OTUs will be divided into two groups, with or without over-dispersion, using a score test^186^ or bootstrap parametric test.^187^ Then, OTUs, without and with over-dispersion, will be modeled using zero-inflated Poisson (ZIP) and ZINB distributions, respectively. RioNorm2 method works best in the case of absolute abundance (count) over the relative abundance (i.e., proportion). The benefits of using RioNorm2^71^ are: 1) It takes account of under-sampling and over-dispersion of data. 2) It consistently yields high power while controlling the FDR, and is robust with small to medium effect size, and different library size and sample size. However, RioNorm2 also has limitations: 1) Like GMPR, since the ratio or log ratio is undefined, RioNorm2 only uses nonzeros in calculating the variance of log-ratio. The appropriateness of excluding zero values is difficult to be evaluated. 2) Only calculating the pairwise dissimilarity among the top abundant OTUs (i.e., the OTUs observed in at least 80% of samples with average count larger than 5 is recommend to be kept for constructing the network of OTUs) is obviously a practical but arbitrary procedure. 3) The fixed threshold value of 0.03^th^ quantile of dissimilarity distribution in search for invariant OTUs for an edge connecting two OTUs needs to be further validated. RioNorm2 is implemented with the R package RioNorm2. The performance of RioNorm2 was demonstrated by a study on microbiome and metastatic melanoma to find the relationship between the microbiome species and the cancer treatment efficiency.^19^

##### Cube root(cbrt)-based data normalization and normalization method aggregation

To improve deep neural network (DNN)-based classification of CRC using gut microbiome stool sample data, Mulenga et al.^72^ proposed a cbrt-based data normalization method and a method that combines a feature extension technique, based on aggregating data normalization methods with data augmentation. The proposed method aims to reduce the effect of dominant features, which is achieved through two steps: 1) It first rescales data points in the data set by multiplying each of them by the standard deviation of the entire raw data set, and then the cube root on the product is computed. 2) It combines the individual normalization methods to extend features of a data set through a data augmentation technique to synthetically produce and combine new samples with the original data^188^ and hence to reduce data variability (i.e., variations in the abundance of a specific gene across samples).

It was demonstrated^72^ that: 1) Aggregation of the normalization methods can improve the classification performance of a DNN model. 2) Combining normalization with data augmentation leverages the strength of the combined normalization methods and provides robust modeling results, although the proposed cbrt method does not outperform other single normalization methods all the time. However, this method also has limitations: 1) It results in significantly increased features in the transformed dataset. The quality is obviously arguable. 2) It also poses the challenge to carefully select the normalization methods to be used in the data transformation technique.^72^ Therefore, users find it difficult to apply the proposed method to their real data. The proposed cbrt-based data normalization and the normalization method by aggregation were reported to improve CRC classification performance of Deep Neural Network (DNN) algorithms.^72^ The integration of feature engineering and data augmentation for CRC identification was also discussed in the study on deep learning-based fusion model for biomedical image classification.^189^

## Evaluation of normalization methods in 16S RNA-seq and shotgun metagenomic data

Most normalization methods work best with their accompanying (i.e., built-in) statistical models or tests and their targeting data, while having worse performances using other statistical models or tests or different data. This scenario is a major common limitation of the so far proposed normalization methods. The performances of most normalization methods were evaluated based on visualization effects, by clustering, ordination techniques and differential abundance analysis. Here, we describe how these normalization methods have been evaluated, and comment on the questions behind them.

### Is normalization necessary?

Does microbiome data really need to be normalized by an independent procedure? This question is related to another question: whether count-based approach or compositional (relative)-based approach, which are the two largely available approaches in microbiome literature, is more appropriate for analysis of microbiome data? Usually, the compositional (relative)-based approach is preferred to relative-based or compositional methods, such as ALDEX2,^162^ and ANCOM^98^ which prefers to use a data transformation or normalization method to normalize microbial taxa abundances. In contrast, the count-based approach, such as edgeR-TMM^121^ and DESeq-RLE,^127^ include an offset (a built-in normalization procedure) in their count models to adjust for the library size (sequencing depth). Some count-based methods advocates^190^ showed that transforming the microbiome count data could potentially decrease the power in detecting significant taxa.

### Is rarefying necessary?

As a normalization procedure, rarefaction resamples an OTU(ASV/gene) table such that all samples have the same sequencing depths. Are corrections for unequal library sizes really needed to be applied? In 16S RNA-seq literature, this topic has been evaluated by McMurdie and Holmes,^40^ Weiss et al.,^46^ and McKnight et al.^54^ However, there is no consensus regarding whether it is appropriate or not to be used in microbiome data. Some researchers still think rarefaction is an effective normalization method.^46,54^ In 2005, rarefying was first recommended for microbiome counts to attenuate the sensitivity of the UniFrac distance^191^ to library size, and later, especially to differences in the presence of rare OTUs.^192^ In microbiome literature until 2013, microbiome researchers often start their data analysis with an ad hoc library size normalization procedure, so-called rarefying by random subsampling without replacement.^40,193–195^ Particularly, although in general, both rarefaction and the size factor-based methods (e.g., TSS, edgeR-TMM, DESeq2-RLE, CSS, and GMPR) have their own weaknesses and strengths for particular applications,^66^ compared to the size factor-based methods, rarefaction is useful and is recommended for alpha- and beta-diversity analysis, especially for unweighted measures and for confounded scenarios, where the sequencing depth correlates with the variable of interest.^46,66^ The rationale is that most taxa in microbiome data are low abundant and their presence/absence strongly depends on the sequencing depth; in such cases, rarefaction can ensure comparison of alpha- and beta-diversity on an equal basis. In contrast, the size factor-based normalization cannot handle this issue. Other researchers^54^ made similar points and recommended that community-level comparisons should generally use proportions (preferably) or rarefied data rather than use methods for variance stabilization transformations (e.g., UQ, CSS, edgeR-TMM, and DESeq-VS) because these methods involve a log transformation that distort communities and alter species evenness.

However, other researchers do not advocate using rarefaction as a normalization procedure.^48,66^ Rarefying procedure throws away sequences from the larger libraries so that all have the same, smallest size, which not only often discards samples that can be accurately clustered by other methods, but also results in a high rate of false positives in tests for differentially abundant species across samples.^40^ Therefore, rarefying biological count data is statistically inadmissible.^40^ There are several reasons for this: First, rarefying (rarefaction) wastes or loses available valid data but does not equalize variances; instead of inflating the variances in all samples, it adds noise (artificial uncertainty) by random subsampling. Second, rarefying (rarefaction) was previously often used in the macro-ecology and early microbiome studies, where their principal objective is to explore or descriptively compare species or samples from different environmental/biological sources. However, current microbiome study has moved beyond exploratory/descriptive comparison of microbiome samples to multivariate analysis of microbiome, in which sample covariates can be adjusted for sample-wise distance matrices. Thus, as a normalization procedure, “rarefying to even sampling depth” is not necessary or is not important for detecting differential abundance of OTUs/ASVs between different samples. Third, rarefying or using rarefied counts is not optimal for downstream clustering analysis and does not improve the sensitivity and specificity of differential abundance analysis of microbiome data. In summary, rarefying or using rarefied counts was evaluated not only with performance costs in both sample-clustering and differential abundance analysis, but also with increased Type-I and Type-II errors.^40^

Rarefying has also been commonly used in shotgun metagenomic data.^195–197^ However, it was shown^91^ that rarefying had a relatively low performance for normalization of metagenomic gene abundance data, both in terms of true positive rate (TPR), false positive rate (FPR), and the ability to control the FDR, as well as the resultant inflated gene-gene correlation. In contrast, increasing the number of DNA fragments will increase the ability to correctly identify differentially abundant genes (DAGs).^91^ Therefore, Pereira et al.^91^ recommended avoiding the use of the rarefying method to correct the differences in sequencing depth for the identification of DAGs.

In summary, although generally rarefying/rarefaction holds promise as a reliable method for comparing microbial diversity or community-level comparisons, when interpreting rarefying, we should consider two points:^196^ 1) rarefaction is used to compare observed richness among samples for a given level of sampling effort rather than attempt to estimate true richness of a community; 2) rarefaction analyses require larger samples; so rarefaction on small samples may yield the incorrect order of the true richness of the sample. Additionally, the concept of rarefying or rarefaction originated in macro-ecology. It may be suitable for exploratory study of macro species, but may be not necessary or equally important in microbiome study. In microbiome study, alpha diversity tends to be less important than beta diversity and functional study. Thus, the methods or models that can address heterogeneity of data and studies to detect core microbial taxa and integrate multi-omics data are more important. Therefore, we weigh generalized models over rarefying or rarefaction to mitigate heterogeneity of data and studies, and recommend using generalized linear mixed models and multivariate analysis for microbiome study.^38,198–201^

### Does variance stabilization matter in normalization?

#### Mitigating variability matters in normalization

Variability in microbiome study includes variations in the abundance of a specific gene across samples due to data sequencing^202^ and heterogeneity of studies such as batch effects.^203,204^ High variability can result in reducing the sensitivity of a model and challenge the data integration and meta analysis to provide convincing analysis results.^202,204–206^ Thus, mitigating variability via normalization matters.

#### Negative binomial (NB) versus poisson model?

Rarefying has some rooting in Poisson model, while the question of variance stabilization is directly relevant to whether the Poisson model or the NB model is more appropriate for the microbiome data. The appropriateness of using variance stabilizing transformations to detect DAGs highlights that the dependence between the mean and variance is not trivial. In contrast to the Poisson model, the NB model can use variance stabilizing transformations to decouple the dependence between mean and variance.^207^

McMurdie and Holmes,^40^ based on their evaluation results and well-established statistical theory, advocated that microbiome investigations should avoid using rarefying altogether. Instead, they advocated that microbiome investigations should use the relevant variance stabilizing transformations to normalize their microbiome data and should use a hierarchical mixture such as the Poisson-Gamma or Binomial-Beta models to model uncertainty. McMurdie and Holmes’ recommendation^48^ for the potential of normalization techniques of DESeq2^62^ and metagenomeSeq^42,145^ have been recognized and incorporated into QIIME, since version 2.0.^46^

#### Rarefying versus variance stabilization transformations?

In 2017, Weiss et al.^46^ compared six existing normalization methods and differential abundance analyses to raw data (none using any methods) using simulation and real 16S rRNA amplicon sequencing data to evaluate the performance of rarefying and variance stabilization transformations methods. The six evaluated normalization methods are proportion, rarefy, logUQ,^50^ CSS,^42^ DESeq-VS(variance stabilization),^62,127^ and edgeR-TMM(Trimmed Mean by M-Values).^121^ On one hand, Weiss et al.^46^ agreed with McMurdie and Holmes^48^ that 1) when the groups differ substantially, rarefying and most normalization methods are clustering well for ordination metrics, based on the presence or absence; and 2) rarefying results in a loss of sensitivity due to elimination of a portion of available data. On the other hand, Weiss et al.^46^ concluded that rarefying remains a useful technique for sample normalization because it can cluster samples more clearly, according to the biological origin, and rarefying itself does not increase the FDR in differential abundance testing. Instead, Weiss et al.^46^ stated that DESeq2 and other alternative methods for variance stabilization transformations are potentially vulnerable to artifacts due to thelibrary size.

The contrast between rarefying and the methods of variance stabilization transformations actually highlights the issues of how to effectively use sample sizes and appropriate statistical models to deal with the high variability of microbiome data. Both rarefying and the variance stabilization transformations methods have limitations. On one hand, discarding data and using a nonparametric test (e.g., Wilcoxon rank-sum test) could lead to false negatives (lower sensitivity).^46^ The severity of the power decrease caused by rarifying depends upon how much data has been thrown away and how many samples were collected. Thus, a general guideline is needed to guide how to rarefy to obtain the highest depth possible^208^ and a justification should be put forth if the samples are discarded. On the other hand, the methods for variance stabilization transformations (e.g., UQ, CSS, edgeR-TMM, and DESeq-VS) relies on log transformations as a mechanism to standardize variances, i.e., usually via a log_2_ adding a pseudocount. The log transformations are typically used to reduce 1) the skewness of the data and 2) the variability due to the outliers, with the assumption that the log transformation can make data conform more closely to the normal distribution.^110,111^ Log transformations facilitate data analysis by making multiplicative models additive and perfectly removing heteroscedasticity when the relative standard deviation is constant.^109^ However, unfortunately, log transformations have at least three disadvantages:^209^ 1) limited effect on values with a large relative standard deviation; 2) cannot deal with the zero value because log zero is undefined; and 3) tend to inflate the variance of values near zero^114^ although it can reduce the large variance of large values. In microbiome literature, the log transformation approach is also criticized^46,54^ because log transformation cannot stabilize variances or heterogeneity; instead, could result in distorting the Bray-Curtis dissimilarity values to poorly match the original values and hence make the interpretation of data difficult.^54^ However, in contrast to rarefying, the negative binomial(NB)-based models that are implemented in DESeq2^127^ and in edgeR^121^ with RLE normalization were able to accurately and specifically detect differential abundance, regardless of the effect sizes, replicate numbers, and library sizes.^40^ Thus, overall, we can consider the normalization methods, along with their NB-based models in the packages edgeR and DESeq2, as mitigating over-dispersion normalization methods. These two models and the other over-dispersed models have been applied in the analysis of microbiome data.^200,201^

### Total count versus median or upper quartile?

In RNA-seq gene expression data, total counts (TC) can be heavily dominated by the most common genes. Thus, theoretically median (Med) and upper quartile (UQ) methods are more robust than the TC method, and the gene count distributions with the 50^th^ (Med method) or 75^th^ (UQ method) percentile are often used as a scaling factor to replace the sum of TC. Med and UQ methods also have been evaluated as more robust and hence better than the TC method for the normalization of RNA-seq data.^50,97,210^

In early microbiome studies, similar data situations have been considered by some microbiome researches, and Med and UQ normalization methods have been often seen applied in microbiome data. However, due to the controversial results that have been already found in RNA-seq literature and the common belief, early in microbiome studies, Pereira et al.^91^ showed that the TC method not only had overall similar or higher performance than Med and UQ normalization methods but also had an overall higher TPR and a lower FPR. Moreover, when DAGs have unbalanced effects, UQ and quantile-quantile (QQ) methods were unable to control the FDR.^91^ There are several reasons for the TC method overall outperforming the Med and UQ methods as also the disparity between shotgun metagenomic and RNA-seq data. First, the estimated scaling factors of the TC method, and the Med and UQ methods are very highly correlated;^211^ thus they have similar overall performance. Second, the differences in the data structure between shotgun metagenomics and transcriptomics, at least partially contribute to the disparity.^91^ The upper quartile was developed specifically for transcriptomics; thus it is not necessarily suitable for shotgun metagenomic data. Third, the highly biased *P*-values and hence high FPR in the identification of DAGs for shotgun metagenomics using UQ and QQ normalization methods are due to invalid underlying model assumptions.^212^ This is because these models do not incorporate gene-specific variability and hence high over-dispersion has been incorrectly interpreted as biological effects.^63,213^ Thus, the problem of the skewed *P*-value distributions generated by improper normalization cannot be addressed by replacing the parametric model (e.g., the over-dispersed Poisson model) with a nonparametric or a permutation-based method.^91^ The above findings were demonstrated by shotgun metagenomic data. More studies are needed to confirm these results by 16S rRNA data because, compared to the shotgun metagenomic data, 16S rRNA data are more over-dispersed and zero-inflated; hence, are less close to RNA-seq data.

### Does over-dispersion matter in normalization?

Emphasis on variance stabilization transformations highlights the importance of over-dispersion in normalization matters. The statistical theory of rarefied counts is based on a hypergeometric model,^53^ which was originally designed to compare a pair of lanes in RNA-seq data, where the individual gene counts in each lane are assumed to follow a hypergeometric distribution and a pair of lanes are compared using a hypergeometric model. In other words, the hypergeometric distribution assumes that the given lane effect will be absent, after accounting for the different total gene counts in each lane. Under the null of absence of a lane effect, the *P*-values across genes are uniformly distributed and thus a lane effect is the deviation from uniformity. It can be assessed using a QQ-plot, and the gene expression effects can be analyzed via a Poisson model to compare the multiple lanes for a lane effect.^53^

Poisson model is not appropriate for microbiome data because the microbiome data are over-dispersed and zero-inflated. The original DESeq-VS^62,127^ and edgeR-TMM^121^ were proposed in the background of the microarray gene expression study. As discussed above, it is still debatable whether these original NB-based models that were proposed for RNA-seq data are appropriate for microbiome data. However, it is obvious that they are more suitable than rarefying to fit microbiome data because mitigating over-dispersion matters in microbiome data analysis, whereas sample rarefying and proportion approaches are the Poisson based-methods. Thus, whether the sample rarefying and proportion approaches are appropriate is relevant to whether the Poisson model or any other relevant model for modeling uncertainty is appropriate. It is obvious that after removing low-depth samples, including zero counts from sample, the Poisson based-rarefying and NB-based models have no significant difference in clustering, ordination, and differential abundance analysis. However, it does not mean that NB-based models are not better than the Poisson based-rarefying methods.

Through shotgun metagenomic data, Pereira et al.^91^ evaluated that edgeR-TMM^61^ and DESeq-RLE^127^ had the overall best performance, both in terms of TPR and FPR. Especially, they had a higher advantage in the unbalanced case, compared to other methods. At the unbalanced DAGs, only edgeR-TMM, DESeq-RLE and metagenomeSeq-CSS were able to correctly control the FDR or only had a moderate bias when the effects of DAGs are lightly or heavily-unbalanced. In addition, both methods estimated the effect size more accurately and showed high correlations of their estimated scaling factors. Compared to DESeq-RLE, edgeR-TMM, in most cases, had overall better performance in terms of slightly higher TPR and lower FPR, making it the highest performing method in this comparison study. The better performance of edgeR-TMM and DESeq-RLE observed in the shotgun metagenomic data is consistent with previous evaluations on RNA-seq data^51^ and 16S rRNA-seq count data.^40^

Two reasons could contribute to the better performance of edgeR-TMM and DESeq-RLE on differential abundance analysis: 1) these two methods do not explicitly adjust for count distributions across samples, while allowing samples to be different in library composition; and 2) these two methods are built on a NB model so that they are able to model over-dispersion of the data, compared to other scaling methods. Although the NB model was criticized for having very low power in detecting differential abundance for small or medium effect sizes and its overall performance is highly sensitive to the library size,^71^ we think mitigating over-dispersion is the main reason that edgeR-TMM and DESeq-RLE outperform other scaling methods.

### Does zero-inflation matter in normalization?

Accounting for zero values in normalization matters in microbiome study.

#### Microbiome data have many zeros

As we described in Section 2, one of the important and unique characteristics of microbiome data is that microbiome data often have many zeros. Compared to RNA-seq data, microbiome sequencing data are more over-dispersed and zero-inflated.^66,201,214^ Excess zeros in microbiome taxa abundance, especially at lower taxonomic levels or OUT/ASV counts (e.g., genus and species), result in only a few taxa or OTUs/ASVs, often dominating across samples, and any given taxa or OTUs/ASVs presenting in all samples is rare.^36^

#### The issues of zeros are not appropriately addressed in the RNA-seq data-based normalization methods

RNA-seq data-based normalization methods typically calculate the size factor after excluding genes with zero values. For example, edgeR-TMM identifies a trimmed set of genes for each sample without including genes with zero values.^61^ DESeq-RLE does not well define the geometric means of genes for genes with zeros.^127^ When the housekeeping genes is used for normalization, only the housekeeping genes with nonzero expression values are considered.^215^ Thus, using these methods that are specifically designed for normalizing RNA-seq data to microbiome data will exclude OTUs with zero values in calculating the size factor, leading to OTUs with zero values that are not well defined.^66^ Thus, both edgeR-TMM and DESeq-RLE use only a small fraction of the “ubiquitous genes” to calculate the size factor after excluding the genes with zero values. When the “ubiquitous genes” were defined by excluding those genes with zero values in all samples, and excluding most, if not all, differentially expressed genes,^131,215^ there is only a small common fraction of data to calculate the size factor. For example, edgeR-TMM sets a reference sample for the size factor calculation, which restricts the size factor calculation to a specific gene set that the reference sample harbors. DESeq-RLE becomes less stable when the gene data become more sparse and even fails if there are no “ubiquitous genes’’. Because the DESeq method sets the negative values resulting from the log-like transformation to zero, it actually ignores many rare species completely.^46^ Moreover, DESeq normalization method was developed mainly for use with Euclidean metrics. Thus, it was shown that this method does not work well with non-Euclidean measures or ecological metrics (e.g., Bray-Curtis dissimilarity), and will result in misleading results, except when using weighted UniFrac.^46^ DESeq and edgeR-TMM assumes that most microbes are not differentially abundant, and among those differentially abundant microbes, the amount of increased/decreased abundance is approximately balanced.^50^ Because microbial environments are extremely variable, DESeq and edgeR-TMM normalization assumptions are likely not appropriate for highly diverse microbial environments. It was shown that edgeR-TMM and DESeq-RLE normalization methods are only able to normalize a small fraction of the available OTUs in real 16S-rRNA data sets.^66^

In summary, both DESeq and edgeR-TMM normalization methods are not informative and hence are not optimal for microbiome data.

#### The issues of zeros cannot be appropriately addressed by replacing zeros with a small pseudo-count value

In practice, one convenient way to deal with the zero problem and therefore to avoid the zero-inflation problem is to replace zero with a small pseudo-count value. This practical strategy is often conducted in compositional data^98,143,144,216,217^ to ensure log(0) to be defined. Not only in compositional-based approach, other normalization and statistical methods, including DESeq and CSS, logUQ methods, also add a constant or pseudo-count (i.e., one) to the count matrix prior to transformation to avoid log(0) undefined. The pseudo-counts are often generated by a Bayesian model.^218^ However, this is not an ideal way to address the zero problem, regardless of whether the pseudo-count was directly added or generated by a model. There are several reasons: 1) Actually, the appropriateness of replacing zero with a small pseudo-count value is built on the implicit assumption that all the zeros are due to under-sampling.^40^ This assumption is one major limitation of this approach because it does not differentiate between structural zeros and sampling zeros.^214^ 2) There is no clear consensus on how to choose the pseudo-count; and hence it is often chosen very arbitrarily. 3) Log transform is nonlinear. Thus, the choice of pseudo-count is sensitive.^143,144^ 4) The clustering results can be highly influenced by the chosen pseudo-counts.^143^. 5) The Bayesian formulation assumes a Dirichlet-multinomial framework, and hence imposes a negative correlation structure on every pair of taxa.^40,46,98,219^ In summary, by replacing zero values with pseudo-counts, not only is the appropriateness not guaranteed, but it also ignores interpreting the different zero values of microbiome data in terms of appropriate concepts and sources of zeros. Therefore, we should carefully interpret the analysis results when the zero values are replaced.^176^

### Does compositionality matter in normalization?

Some researchers do not consider that microbiome data are compositional or the compositional effects are attenuated due to its larger numbers of taxa, whereas others really think microbiome data are compositional (details in Section 3.4.3). Here, we summarize three main points regarding the mitigating compositionality matters in normalizing microbiome data:

1. The traditional normalization methods are not optimal for compositional data. RNA-seq approach assumes that the majority of genes (in the case of microbiome, OTUs/ASVs/taxa) are not differentially abundant^51^ and the developed models were used to estimate over-dispersion.^42^ RNA-seq approaches,^162^ including the DESeq2 method^46^ and the traditional proportion normalization,^220^ have poor performance in the analysis of compositional data due to high FDR.
2. RNA-seq approach (e.g., DESeq2) was designed to provide increased sensitivity on smaller datasets (<20 samples per group), when the library sizes are larger and/or very uneven; this kind of method tends to increase FDR. And
3. The practice of manually adding a pseudo-count to the matrix prior to DESeq2 transformation also increases the FDR.^46,162^

Put together, from the perspective of compositional analysis, microbiome data should be normalized and analyzed by a compositionally aware method such as ANCOM because it is not only very sensitive (for >20 samples per group) but it is also critical to have a good control over FDR and increase its power.^46^ However, as we reviewed in Section 4.6, compositional-based approach cannot solve the zero problem.

### Are different normalization methods needed for 16s rRNA-seq data and shotgun metagenomic data?

Although rare studies have evaluated this topic, it is certain that normalization methods for both 16S rRNA-seq data and shotgun metagenomic data should be evaluated, based on their unique data characteristics. As we reviewed in Section 2, these two kinds of data share unique data characteristics: over-dispersion, sparse with many zeros, and compositionality and heterogeneity. There are also some differences. For example, shotgun metagenomic data may be less over-dispersed and zero-inflated compared to 16S rRNA-seq data, and may be closer to RNA-seq data.

In 2018, Pereira et al.^91^ evaluated nine normalization methods that are available for high-dimensional count data using the gene abundance data generated by shotgun metagenomic sequencing in terms of: 1) ability to identify differentially abundant genes (DAGs) using an over-dispersed Poisson generalized linear model (OGLM)^212,221^ and 2) correctly calculating unbiased P-values and controlling the FDR. In general, most normalization methods that have been evaluated by the shotgun metagenomic data^91^ have similar performances to previously using RNA-seq data.^50,51^

In summary, most normalization methods: 1) could satisfactorily normalize metagenomic gene abundance data when the DAGs were equally distributed between the groups, while the performance was substantially reduced when the distribution of DAGs were more unbalanced; 2) had a reduced true positive rate (TPR) and a high false positive rate (FPR) as well as was unable to control the FDR. 3) The relative normalization results also had been majorly impacted by the size of the groups with several methods even underperforming when only few samples were present. Particularly, among the nine normalization methods: 1) The edgeR-TMM and DESeq-RLE overall had the highest performance, which are therefore recommended for the analysis of gene abundance data;2) CSS also showed satisfactory performance when sample sizes are larger; 3) Normalization using quantile-quantile, median and upper quartile, as well as rarefying the data, overall had the lowest performance, resulting in high FPRs and in many cases FPRs reached unacceptable levels. Thus, using quantile-quantile, median, upper quartile, and rarefying to normalize metagenomic gene abundance data was not recommended by Pereira et al.^91^

Overall, Pereira et al.^91^ demonstrated that improper methods may result in unacceptably high levels of false positives and hence lead to incorrect biological interpretation. Thus, this study highlighted the importance of selecting the suitable normalization methods in the analysis of data from shotgun metagenomics.

### Does evaluation method matter in evaluating the performance of normalization?

To evaluate the performance of normalization, generally five kinds of evaluation methods have been used: 1) receiver operator characteristic (ROC) curve; 2) correlation and association analyses; 3) sample-wise distance or dissimilarity metrics; 4) clustering and ordination techniques; and 5) statistical tests.

#### ROC curve

ROC curve^222,223^ is a visual representation of the diagnostic capability of binary classifiers. It is usually used to reveal the sensitivity – true positive rate (TPR) and specificity (1 – false positive rate (FPR)). ROC curve is most often used to evaluate statistical and machine learning methods including normalization methods.

#### Correlation and association analyses

The performance of normalization methods are also often evaluated by Pearson’s correlation analysis^224^ and Spearman’s correlation analysis,^225^ intersample variability,^60^ Matthews correlation coefficient (MCC),^226^ and intraclass correlation coefficients (ICC).^227^

#### Sample-wise distance or dissimilarity metrics

Sample-wise distance/dissimilarity measures are often used to measure the performance of normalization. Bray-Curtis dissimilarity,^228^ which has one favorable nature of not requiring equal variances, is most commonly used in microbiome studies. Other distance/dissimilarity metrics also have been used in microbiome studies including binary Jaccard, Euclidean distance to measure each OTU as a dimension, Poisson distance to measure sample-wise distance implemented in the PoiClaClu package,^229^ mean squared difference of top OTUs implemented in edgeR,^121^ unweighted UniFrac distance,^191^ and weighted UniFrac distance.^230^

#### Clustering and ordination techniques

Clustering and ordination are used along with sample-wise distance/dissimilarity metrics. The four most commonly used hierarchical clustering analysis (HCA) methods are single linkage,^231^ complete linkage,^232,233^ average linkage.^234^ and Ward hierarchical grouping.^235^ Among the ordination methods, principal component analysis (PCA),^236–238^ principal coordinate analysis (PCoA),^239^ nonmetric multidimensional scaling (NMDS),^240–243^ and especially PCoA are most often used in microbiome literature.

#### Statistical tests

Various statistical tests have been used to evaluate normalization methods in various proposed methods for normalization of microbiome data, including parametric Welch *t*-test implemented in the packages phyloseq and multtest,^244^ nonparametric Wilcoxon rank-sum test (or Mann-Whitney *U* test), the differential abundance analysis, and particularly edgeR-exactTest,^121,245^ DESeq-nbinomTest,^127^ DESeq2-nbinomWaldTest,^62^ Voom,^138^ metagenomeSeq,^42,145^ ANCOM,^98^ and multivariate analysis, such as PERMANOVA.^246^

Usually in microbiome studies, one or more of above five categorical criteria are often combined together to evaluate normalization methods. The effects of normalization from the proposed or compared normalization methods are evaluated based on the performances of standardizing within-sample variance across samples, data separation in clustering and/or in ordination plots between groups and in differential abundance analysis.^40,42,46,50,97,247^ Although the microbiome normalized data evaluated by different normalization methods have different qualities and could lead to different conclusions on the evaluated normalization methods, and different downstream statistical results could be obtained when using the microbiome data normalized by different normalization methods, some preliminary conclusions on the performance of normalization evaluation can be achieved.

***1) overall RNA-seq data-based normalization methods have better performances than ecology data-based and traditional normalization methods***. In general, CSS, DESeq-VS, and edgeR-TMM normalization methods overperform the proportions and rarefying in normalizing 16S rRNA-seq data.^40^ and shotgun metagenomic sequencing data.^54^

***2) due to targeting unique microbiome data characteristics, overall microbiome data-based normalization methods have better performances than RNA-seq data-based as well as ecology data-based and traditional normalization methods.*** It was shown^154^ that the specifically designed normalization methods for microbiome data including wrench edgeR-TMM (also using wrench normalization), wrench Hurdle, ANCOM-BC are clustered together but are separated from the methods designed for RNA-Seq data, which are clustered together including CSS, CPM, DESeq-VS. Other study^155^ also demonstrated that metagenomeSeq-wrench normalization method had controlled FDR well across settings, while maintaining decent power. This suggests that the normalization methods that are specifically designed to handle specific biases associated with microbiome data is important.

***3) any normalization methods including microbiome data-based and RNA-seq data-based normalization methods cannot simultaneously address all the issues caused by microbiome unique data characteristics in terms of controlling FDR while maintaining power and flexibility.*** None of the normalization methods can address all the challenges of microbiome data. This has been demonstrated in so far the most comprehensive evaluation of microbial differential abundance analysis(DAA) methods.^155^ This study evaluated most available microbial DAA methods with their default normalization methods including Aldex2, ANCOM-BC, metagenomeSeq, DESeq2, edgeR-TMM, Omnibus test (using GMPR normalization), and RAIDA, and found that none of the DAA methods are able to be simultaneously robust, powerful, and flexible. Here, we briefly summarize some main arguments:^155^ 1) The DAA methods that explicitly address compositional effects including ANCOM-BC, Aldex2, and metagenomeSeq did have reduced false-positive rates, but they are still not optimal due to Type I error inflation or low statistical power. 2) ANCOM-BC, Aldex2, and Omnibus test did outperform the TSS-based methods in FDR control, but their performances are still not satisfactory for strong compositional effects. 3) Both ANCOM-BC and Omnibus test did not work well under a small sample size. 4) Some other methods offered the best FDR control, but at the cost of low power and especially for rare taxa. 5) Most methods failed to control the FDR when the sequencing depths differed cross groups, indicating rarefaction may be still required for these methods.

***4) proportions and rarefying methods may be still suitable for conducting comparisons of entire communities, but are not important for microbial function study.***The roles that proportions and rarefying methods play in microbiome study are still controversial. For ecological reasons or perspective, and specifically when comparing communities, three measures have been considered as important:^54^ 1) fully standardize reads in using the Bray – Curtis (BC) dissimilarity and other distance and dissimilarity metrics to measure beta diversity; 2) the species evenness; and 3) the community functionality of dominant species.

On one hand, prior to calculating distance or dissimilarity measures, proportions or rarefying may be the most suitable methods for transforming ecological data to produce accurate comparisons among entire communities (i.e., beta diversity).^54^ On the other hand, the UQ,CSS, edgeR-TMM, and DESeq-VS methods have potential problems in calculating all the above three measures to compare communities.^54^ Here, three arguments can be summarized: 1) These normalization methods (e.g., UQ, CSS, edgeR-TMM, and DESeq-VS) do not guarantee equal number of reads across samples, which may raise serious concerns about their applicability for community-level comparisons. 2) These normalization methods focus on standardizing the within-sample variance across samples,^97,247^ which suppress differences in species evenness. 3) these methods relie on log transformations as a mechanism to standardize variances, i.e., usually via a log_2_ adding a pseudo-count. The behind algorithm is to reduce the effect of highly abundant OTUs so that the effects of rare OTUs (species) can be detected. Given both rare OTUs (species) and the dominant OTUs (species) in an ecological community can play important functional roles, reducing the importance of dominant OTUs (species) and amplifying the importance of rare OTUs(species) may provide misleading insight into the differences among communities. However, as we discussed in above Sections 4.2 and 4.3, microbial function study has been weighted more important than microbial/ecological community analysis, suggesting that detecting whether specific OTUs/taxa differ across groups is a more important topic compared to just describing alpha and beta diversities in current microbiome study.

***5) the basic distinction of statistical analysis between count-based and compositional (relative)-based approaches could also lead to the different evaluation results of normalization methods.*** In general, it is agreed that microbiome data are structured as multiple taxonomic levels and encoded as phylogenetic tree, high-dimensional, sparse, and often have many zeros. However, some microbiome researchers think microbiome data are discrete and real counts should be analyzed using a count-based method, whereas others consider that microbiome data are compositional (relative) and should be analyzed using a compositional (relative)-based approach. When inferencing microbial taxa, both count-based and compositional(relative)-based approaches will face the statistical issues of dependency, sparsity, over-dispersion, and zero-inflation. However, these two approaches weigh the importance of microbiome normalization methods differently for both 16S rRNA sequencing and shotgun metagenomic sequencing data and use different strategies to address these challenges.

The count-based approach advocates consider that the microbiome data are not mainly compositional. Their arguments have been discussed in Xia:^36^ 1) Compared to ecology data, microbiome data usually have large number of taxa and hence have more high dimensions, but the compositional effect on the large diversity of samples is mild or is attenuated if more taxa are included in this study. Thus, the spurious correlation concern due to compositionality that is originated in ecology study is not a big deal. 2) Composition-based approach interprets biological differences based on the ratio of taxa rather than to directly detect which taxa are associated with the outcome of interest. This does not make sense biologically. 3) Microbiome data compositionality may be corrected when the absolute cellular abundances can be estimated as microbiome sequencing data science technologies advance. Overall the issues of overdispersion and zero inflation as well as dependency of taxa are more considered in count-based approach. In the count-based approach, the taxa abundance is normalized using an offset in a standard count model (e.g., zero-inflated and zero-hurdle models) and variations are adjusted via covariates. In contrast, the direct challenges of how to replace the zero values prior to normalization and how to deal with the boundary [0, 1] are mainly faced to be addressed by composition(relative)-based approach.

In summary, several unique characteristics (i.e., multidimensionality, compositionality, sparsity with many zeros, and heterogeneity) exist simultaneously in one microbiome data/study. The real challenges are due to the factor that none of normalization and DAA methods can simultaneously address the issues caused by these unique microbiome data characteristics.

## Conclusions and perspectives

Microbiomes are associated with various gastrointestinal (GI) tract/and non-GI tract cancers. However, the challenge is that microbiome data have unique characteristics, which often make standard statistical data analysis methods less effective. Normalizing microbiome data has been evaluated as a necessary step for statistical analysis microbiome data.

### Limitations of normalization methods

Microbiome normalization methods were initially adopted from other research fields such as microarray gene expression, RNA sequencing, and ecology studies. Later microbiome researchers and statisticians developed their own specifically designed methods to target microbiome unique data characteristics. Adoption of methods from the well-established fields is a good starting point and often a necessary stage to develop more appropriate methods for microbiome data. But the available methods from other fields (“old bottles”) are not necessarily fitted to fill the new microbiome data (“new wines”). Thus, there is a need to develop new normalization methods appropriate for microbiome unique data.

Currently normalization methods in 16S rRNA-seq and shotgun metagenomic sequencing studies are still few and most normalization methods originated in RNA-seq and microarray studies.

The normalization methods developed in 16S rRNA-seq and shotgun metagenomic studies both have limitations. First, although newly specifically designed methods were proposed to deal with microbiome unique data characteristics (e.g., sparsity, compositionality, over-dispersion, and zero inflation), they are often evaluated on weighing one characteristic over another. The development of methods that can be used to address multiple microbiome unique data characteristics is still challenging. Second, the overall lowest performance of quantile-quantile, median and upper quartile normalization methods, and rarefying in general have been recognized. However, this method and especially rarefying are still advocated by some microbiome researchers. Rarefying originated in rarefaction, in which Poisson modeling and ecology sampling are used. Based on our experience on 16S rRNA-seq and shotgun metagenomics studies, rarefaction on the one hand can facilitate comparisons of alpha- and beta-diversity, but on the other hand results in loss sensitivity due to elimination of a portion of available data. When the normalization methods were applied in cancer research (and also in other research fields), usually the advantages and disadvantages of these methods were not mentioned or recognized. For example, the concept of rarefication was adopted from macro-ecology; however, its limitation was rarely discussed when it was applied. Third, normalization of microbiome data is a complicated topic. Many sources of unwanted variation can affect read counts, such as multiple library preparation protocols, sequencing platforms, sequencing technology (e.g., read length, paired- versus single-end reads). Most currently proposed methods focus on addressing one of the microbiome unique data characteristics and/or within one dataset. The methods that aim to move beyond only adjusting for one simple difference such as the sequencing depth and therefore are able to adjust for other often unknown and more complex effects are expected.

### Challenges and future directions

No large differences between microbiome cancer research and other microbiome research fields have been identified so far. We noticed that a few normalization methods were developed using microbiome cancer data. But few studies have clearly described whether or not there are differences between cancer microbiome data and other microbiome data. Therefore, we can expect that normalization methods that were developed in other microbiome data can be applied into microbiome cancer study and the methods that were developed based on microbiome cancer data can also be employed in other microbiome studies. The critical point is that whether these methods are fitted to the microbiome data to address the unique data characteristics such as to mitigate the sparsity, overdispersion and zero inflation, as well as other heterogeneities and unwanted biases.

While the overall normalization methods have been evaluated to be necessary for both 16S rRNA-seq and shotgun metagenomic data and some of the newly specifically designed methods are promising, their applications in real microbiome studies are still challenging. We expect more works to be done for evaluating these available methods and developing new specifically designed methods to target unique characteristics of microbiome data.

However, challenges remain in microbiome research field. On the one hand, much work still needs to be done to characterize microbiome data such as what are the differences and their respective advantages and disadvantages between 16S rRNA and shotgun metagenomic data? What roles will microbiome normalization methods play in 16S rRNA and shotgun metagenomic data? Whether or not different normalization methods are required when microbiome data were generated by 16S rRNA and shotgun metagenomic sequencing techniques? What total number of sequenced reads (or sampling depths) should always be considered when evaluating sequencing data, regardless of method used to generate it?^33^ Or do16S rRNA and shotgun metagenomic sequencing methods require different sequenced count reads to obtain effective statistical analysis? Generally, compared to the data generated by 16S rRNA sequencing method, the unique data characteristics of shotgun metagenomic data are closer to RNA-seq data. It was reviewed^248^ that analysis of RNA-seq data does not require “sophisticated normalization.” The question is whether it is true for shotgun metagenomic data? Whether deeper sequencing is better or some levels of sequencing are sufficient for effective statistical data analysis to provide insight into microbiome differences between groups. On the other hand, we are sure that several microbiome unique characteristics simultaneously exist in one dataset; thus one cannot rely on one method to address or correct all the heterogeneities or biases. Hence, integrating strategy may be a good approach such as through accommodating multiple potential normalization methods in microbiome association analysis.^249^ However, we need to keep in mind that the more appropriate methods should be based on study design and the heterogeneities and biases that specifically dominate the data and hence need to be addressed and corrected.
